# The asymmetric list shift effect – flexible adaptation to new context demands?

**DOI:** 10.3758/s13414-026-03284-x

**Published:** 2026-06-09

**Authors:** Kathrin Treittinger, Shu Yang, Rico Fischer, Gesine Dreisbach

**Affiliations:** 1 University of Regensburg, Universitätsstraße 31, 93053 Regensburg, Germany; 2 University of Greifswald, Greifswald, Germany

**Keywords:** Cognitive control, Control adaptation, Proportion congruency, Practice

## Abstract

The list-wide proportion congruency effect describes how the congruency effect varies depending on the frequency of incongruent trials within a block. Specifically, the congruency effect is larger in mostly congruent (MC) blocks compared to mostly incongruent (MI) blocks. Research has shown that adaptation to these blocks does not change symmetrically when transitioning between them: moving from MC to MI leads to a rapid decrease in the congruency effect while transitioning from MI to MC results in little or no increase (Abrahamse et al., 2013). We aimed to investigate this asymmetric list shift (ALS) effect in a within-participants design where all participants experienced both transitions (MC-MI and MI-MC). Throughout Experiments 1–3 using a color-word Stroop task, we identified various forms of practice – including trial-type, stimulus-specific learning, general RT decrease, and a decrease in congruency effects over time (see Schmidt, 2016) – that can either facilitate or obscure the ALS effect, depending on the order in which participants experienced the transitions. In Experiment 4, using a face-name version of the Stroop task, where we minimized practice-related confounding factors by employing more complex stimuli and used a new stimulus set in the second transition, an ALS effect was observed regardless of the order of transition and for frequency-unbiased (i.e., 50% congruent) items. The role of practice effects and implications for the (in)-flexibility of control adaptations will be discussed.

**Supplementary Information:**

The online version contains supplementary material available at 10.3758/s13414-026-03284-x.

## Introduction

In everyday life, we constantly adapt our behavior to meet changing environmental demands. For example, consider a driver who regularly travels a specific road through a forest known for deer crossings. This driver instinctively adapts their level of control, becoming more focused or relaxed depending on the time of day and the season. During bright daylight, the driver might be more relaxed, with their mind free to wander. However, as dusk approaches, or in autumn when deer are more active, they automatically shift to a more focused driving style – frequently scanning the roadside, easing off the accelerator, and preparing to brake if necessary. Successfully managing this transition between relaxed and focused driving requires cognitive control, an umbrella term for the higher-order processes that enable a response to the demands of a dynamic environment (Cohen et al., 2007; Miller & Cohen, 2001). Here, we aim to further investigate the flexibility of control adaptations, questioning whether individuals can genuinely shift their control mode based on the demands of different situations that require distinct optimal strategies or if they continue using outdated yet once-effective processing strategies.

In cognitive psychology, evidence for cognitive control is often assessed using conflict paradigms, such as the Stroop task (Stroop, 1935), the Flanker task (Eriksen & Eriksen, 1974), and the Simon task (Simon, 1969). These classic paradigms typically involve stimuli with multiple dimensions, some relevant to the task and others irrelevant (see Kornblum et al., 1999, for a detailed taxonomy of dimensional overlap). For example, in Stroop’s (1935) color-naming task, participants are asked to identify the print color (i.e., the relevant dimension) of a word that itself represents a color (i.e., the irrelevant dimension). The print color can either be congruent (e.g., *RED* printed in red) or incongruent (e.g., *RED* printed in green) with the word’s semantic meaning. Because word reading is generally faster and more automatic than color naming, congruent color words can facilitate the color-naming task (e.g., Brown et al., 2002; MacLeod, 1991). In contrast, during incongruent trials, where the meaning of the word interferes with the print color, cognitive control mechanisms are required to suppress the automatic tendency to read the word and instead focus on naming the color (Miyake et al., 2000). As a result, responses during incongruent trials are typically slower and more error-prone than those in congruent trials (for a review, see MacLeod, 1991). These performance differences between congruent and incongruent trials, known as the *congruency effect*, reflect the influence of irrelevant information and the behavioral costs associated with exercising cognitive control (e.g., Braem et al., 2019; Shenhav et al., 2013).

While congruency effects are often observed, research has shown that their magnitude can be influenced by manipulating *proportion congruency* (PC), which refers to the frequency of conflict within a specific context (for reviews, see Bugg, 2017; Bugg & Crump, 2012). This manipulation was initially investigated using the *list-wide proportion congruency* (LWPC) paradigm, where the proportion of congruent and incongruent trials is varied across two blocks (or lists; Logan & Zbrodoff, 1979). One block consists mostly of congruent (MC) trials, typically containing 67–80% congruent and 33–20% incongruent trials, reflecting a sustained low-conflict context (see Bugg & Crump, 2012). Conversely, the other block consists mostly of incongruent (MI) trials with the reverse ratio, indicating a sustained high-conflict context. Generally, the congruency effect is significantly reduced in MI blocks compared to MC blocks (e.g., Bugg et al., 2011; Kane & Engle, 2003; Lindsay & Jacoby, 1994; Logan & Zbrodoff, 1979; Logan et al., 1984; Lowe & Mitterer, 1982; West & Baylis, 1998).

The LWPC manipulation establishes a rather stable experimental environment that reveals the level of cognitive control needed for most trials in a given context (e.g., Lindsay & Jacoby, 1994; Logan & Zbrodoff, 1979; Surrey et al., 2019). This context stability is assumed to enable the implementation of list-wide adjustments in cognitive control, which are commonly used to explain the LWPC effect (e.g., Braver et al., 2007; but see Mendl et al., 2024). In high-conflict contexts within MI blocks, the constant demand for cognitive control during incongruent trials heightens goal shielding, thereby increasing attentional focus on the relevant color dimension. This sustained attentional selectivity minimizes the impact of the irrelevant word dimension, thereby reducing conflict during incongruent trials and resulting in a smaller congruency effect. In contrast, less cognitive control is needed in low-conflict MC blocks, where word reading is mostly aligned with the correct response. This reduced need for cognitive control and its relaxation leads to slower and more error-prone responses during incongruent trials, thus increasing the congruency effect (see Braem et al., 2019). These list-wide top-down adjustments in attentional control states in response to changing environmental demands (e.g., the PC within blocks) are referred to as control adaptation (e.g., Botvinick et al., 2001; for a review, see Egner, 2017).^1^

Although recent research suggests that humans can flexibly adapt to changing environmental demands, evidence indicates that certain control states can persist, leading to maladaptive behavior. This stuck-in-set phenomenon occurs when individuals continue to rely on a previously effective response strategy that is no longer suitable in a changed context (for evidence of stuck-in-set phenomena in problem-solving tasks, see Luchins, 1942; for similar evidence in response conflict tasks, see Hefer & Dreisbach, 2017). To illustrate, let us return to the deer-crossing scenario mentioned earlier. While driving in shielded control mode, the driver may notice that a fence has been installed to prevent deer crossings, but fails to adapt their behavior fully. As a result, they might continue scanning the roadside, reducing their speed, and preparing to brake, maintaining a heightened level of focus even when it is no longer necessary.

Abrahamse et al. (2013) examined whether shifting from an MC block to an MI block produces effects like those observed when shifting in the opposite direction. In their Experiment 1A, the authors employed a standard LWPC manipulation in a Stroop paradigm, dividing participants into two groups: One group began with an MC block followed by an MI block (i.e., MC-MI), while the other group started with an MI block before transitioning to an MC block (i.e., MI-MC). Their results revealed a pronounced asymmetry in the magnitude of the change in congruency effects depending on the direction of the shift. Participants in the MC-MI transition, who were initially trained on primarily congruent items, demonstrated a fast and strong adaptation to the subsequent high-conflict context, marked by a noticeable decrease in the congruency effect in response to more incongruent trials. In contrast, participants in the MI-MC transition, who started with mostly incongruent items, showed little to no adaptation when moving to the subsequent low-conflict context. Despite encountering a higher proportion of congruent trials, the congruency effect did not significantly increase, suggesting limited adaptation to the less demanding context (Abrahamse et al., 2013).

A straightforward interpretation of this asymmetry is that the frequent occurrence of incongruent trials leads to increased goal shielding, as the attentional focus on the relevant color dimension helps minimize interference. However, this shielding control state not only reduces the congruency effect but also prevents participants from realizing that the need to shield against conflict is no longer necessary in the subsequent low-conflict context. As a result, the persistent shielding impairs their ability to adapt to the new context and take advantage of the change in PC (Abrahamse et al., 2013). Overall, Abrahamse et al.’s (2013) findings highlight an asymmetry in control adaptation, with a significantly larger decrease in the congruency effect during the MC-MI transition compared to the observed increase during the MI-MC transition. This phenomenon, referred to as the *asymmetric list shift* (ALS) effect, has been argued to demonstrate the inflexibility of control adaptations under certain contextual conditions.

Alternative accounts attribute the ALS effect to practice-related potential artifacts. Improvements in response times (RTs) over the course of an experiment are well established and typically reflect general practice effects (Logan, 1988; Schmidt, 2016). Beyond this general RT decrease over time, it has been argued that congruency effects also tend to diminish with practice, likely because participants improve more on initially difficult incongruent trials (Schmidt, 2016). The combination of different practice effects within a given transition (MI-MC or MC-MI) could potentially mimic the ALS effect (Schmidt, 2016). More specifically, during the MC-MI transition, both the increased frequency of incongruent trials and the general decrease in congruency effects observed with practice predict a reduction of the congruency effect. In contrast, during the MI-MC transition, the shift to more frequent congruent trials should increase the congruency effect, which, however, is counteracted by the general decrease in congruency effects observed with practice, potentially reducing the congruency effect (see Schmidt, 2016). Thus, practice effects may reinforce each other in the MC-MI transition but cancel each other out in the MI-MC transition, which could account for the observed asymmetry. Indeed, Schmidt’s (2016) re-analysis of the ALS effect reported by Abrahamse et al. (2013) highlighted the critical role of practice: responses were faster, and the congruency effect was smaller in the second block, providing clear evidence for practice effects. At the same time, residual effects suggest that practice may not fully account for the phenomenon, leaving room for other mechanisms, such as conflict adaptation (Schmidt, 2016).

In the present study, we further investigated the inflexibility of control adaptations, questioning whether the ALS effect would persist when participants experienced both transitions. We thus directly tested whether prior experience with one transition type (e.g., MC-MI) would influence adaptation during the subsequent opposite transition (e.g., MI-MC). Within the LWPC manipulation, the present study therefore examined whether participants, after experiencing a strong shift in attentional focus during the MC-MI transition, would still fail to relax the shielding control state when moving back to a low-conflict context in the subsequent MI-MC transition, or whether experience with the initial shift from a low-conflict to a high-conflict context would enable them to detect the change and flexibly adjust their control settings (i.e., possibly attenuating or eliminating the ALS effect). Conversely, if the ALS effect truly reflects a rigid, high-conflict-induced shielding state, then starting the experiment with an MI-MC transition might cause this shielding mode to persist throughout, again potentially limiting strong adaptation during the subsequent MC-MI transition (i.e., possibly attenuating or eliminating the ALS effect).^2^ Returning to the deer-crossing analogy, we wanted to know whether participants would continue to rely on the shielding control state, even after learning that deer are far less likely to appear in fenced areas than in unfenced ones. To this end, we modified Abrahamse et al.’s (2013) original between-participants design and employed a within-participants approach instead. We asked whether adaptation would remain faster and stronger in the MC-MI transition compared to the MI-MC transition, or whether prior experience with one type of transition would attenuate or even eliminate the asymmetry altogether. Given that the combination of different practice effects within a given transition (MI-MC or MC-MI) could potentially mimic the ALS effect (see Schmidt, 2016), it is essential to consider practice-related influences. Because such effects were already accounted for in a between-subjects design, their impact might be even stronger in a within-subjects design. Therefore, throughout the experimental series, efforts were made to control for practice-related confounding factors.

## Experiment 1

Experiment 1 consisted of two experimental sessions. That is, all participants performed both transitions from a PC1 block (MC or MI) to a PC2 block (MI or MC): They switched from MC to MI in one session and from MI to MC in another session, with the order of transitions counterbalanced across participants. In line with Abrahamse et al. (2013), we expected a significant ALS effect with a stronger adaptation in the MC-MI transition compared to the MI-MC transition.

### Method

#### Participants

Experiment 1 was initially conducted as a pilot study. The sample size was therefore roughly based on Experiment 1A in Abrahamse et al. (2013), which included only 20 participants in a between-subjects design. Since we employed a modified version of the task in an extended within-subjects design and aimed to ensure sufficient power to detect a comparable effect, we decided to increase the sample size by a factor of 2.5. A total of 53 participants took part in Experiment 1. However, data from three participants were excluded from further analyses because they did not participate in the second experimental session. Thus, the final sample consisted of 50 participants (43 females; *M*_*age*_ = 24.56 years, *SD* = 3.56), 92% of whom were students. Participants were recruited from the University of Greifswald and received partial course credit for their participation. Data collection took place between March 2 and March 23, 2022. Participants were native German speakers with normal color vision. All participants in the present and subsequent experiments provided informed consent in accordance with the ethical standards of the national research committee and the 1964 Helsinki Declaration (and its later amendments).

#### Apparatus and stimuli

The experiment was conducted as a computer-based online study, which prevented standardization of equipment details (note: all sizes are expressed in height units, relative to the height of the browser window). The color-word Stroop task was programmed using PsychoPy (v2021.2.3; Peirce et al., 2019, 2022), and data collection was performed using the open-source platform Pavlovia.org (see also Bridges et al., 2020).

The Stroop stimuli consisted of four German color words (in English: *RED*, *BLUE*, *YELLOW*, and *GREEN*) presented in the corresponding print colors from PsychoPy’s standard palette (“red,” “blue,” “yellow,” and “green”). The words were presented in Arial font (letter height 0.05), centered on the screen against a gray (“gray”) background. The task followed a four-alternative forced-choice (4-AFC) paradigm, divided into two alternating subsets with non-overlapping stimulus and response sets (subset 1: RED and BLUE; subset 2: YELLOW and GREEN; e.g., the word RED never appeared in yellow; see Blais & Bunge, 2010; Bugg et al., 2008; Schmidt, 2014). This design ensured that each color word was presented either in its respective congruent print color (e.g., RED in red) or in a predetermined incongruent print color (e.g., RED in blue), resulting in a total of eight unique stimuli drawn from the full 4 × 4 stimulus matrix. Participants responded to the stimuli using color-specific keys on the keyboard. Across trials, the keys C, V, N, and M were consistently mapped to the colors red, blue, yellow, and green, respectively. Responses were made using the middle (C, M) and index (V, N) fingers of both hands, with each subset of stimuli uniquely assigned to either the left or the right hand.

#### Procedure

The experiment consisted of two sessions conducted on separate days, with no more than 4 days in between. Each session consisted of two practice blocks followed by two test blocks representing one of two possible transitions from a PC1 block (MC or MI) to a PC2 block (MI or MC). The order of these transitions was counterbalanced across participants, with one half experiencing the MC-MI transition in the first session and the MI-MC transition in the second session, while the other half received the reverse order. Participants were not explicitly informed about the changes in transitions and blocks between sessions.

To familiarize participants with the color-specific key mapping, each session began with 24 practice trials (six trials per color) in which participants responded to colored squares. This was followed by a second practice block consisting of 48 Stroop task trials (six trials for each color-word combination) to help participants become accustomed to the experimental task. Each session then proceeded with two test blocks of 200 trials each, with the proportion of congruent trials varying across experimental conditions. Participants were allowed a brief, self-paced break between blocks. In the MC blocks, 80% of the trials were congruent (160 trials, 40 per color), while 20% were incongruent (40 trials, ten per color), resulting in a list-wide PC of 80% (i.e., PC-80). In contrast, the MI blocks had the opposite ratio (i.e., PC-20). The order of stimulus presentation within each block was pseudorandomized to prevent direct repetition of subsets in consecutive trials. This design ensured that stimuli were not repeated and required participants to alternate their responding hand on each trial. The specific proportions of congruent and incongruent trials in the MC and MI blocks are summarized in Table 1.^3^

**Table 1:** Color-word combinations in percentages for Experiments 1, 2, and 3

Exp	PC	r/R	r/B	b/B	b/R	y/Y	y/G	g/G	g/Y	p/P	p/M	m/M	m/P
1	MC	19.48	5.08	20.24	5.20	20.23	4.88	20.05	4.84	-	-	-	-
	MI	5.03	20.30	5.08	19.60	4.95	20.34	4.96	19.76	-	-	-	-
2	MC	19.86	5.00	20.08	5.07	19.98	5.12	20.09	4.82	-	-	-	-
	MI	4.96	20.16	5.08	19.80	4.92	19.62	5.04	20.41	-	-	-	-
	50/50	12.94	12.54	12.28	12.23	12.49	12.49	12.29	12.73	-	-	-	-
3	MC	16.95	4.24	16.95	4.24	16.95	4.24	16.95	4.24	3.81	3.81	3.81	3.81
	MI	4.24	16.95	4.24	16.95	4.24	16.95	4.24	16.95	3.81	3.81	3.81	3.81

In Experiment 4, the proportions of each set of face-name combinations corresponded to the percentages of color-word combinations in Experiment 3; Exp. = Experiment; PC = proportion congruency; MC = mostly congruent; MI = mostly incongruent; 50/50 = frequency-unbiased blocks; r/R = red/RED; b/B = blue/BLUE, y/Y = yellow/YELLOW, g/G = green/GREEN, p/P = purple/PURPLE, m/M = magenta/PINK

Participants were instructed to respond as quickly as possible to the print color of the color word while maintaining accuracy. In each trial, a Stroop stimulus was presented and remained on the screen until a response was given. Feedback was provided only for errors, displaying the German word “Fehler” for 1,000 ms. After a 500 ms inter-trial interval, during which the screen went blank, the next trial started. Across both sessions, each participant completed both transitions (MC-MI and MI-MC) in a total of four blocks of 200 trials each, totaling 800 trials per participant. The entire experiment took approximately 25 min, during which response times (RTs) and error rates (ERRs) were recorded.

#### Design

**Fig. 1:**
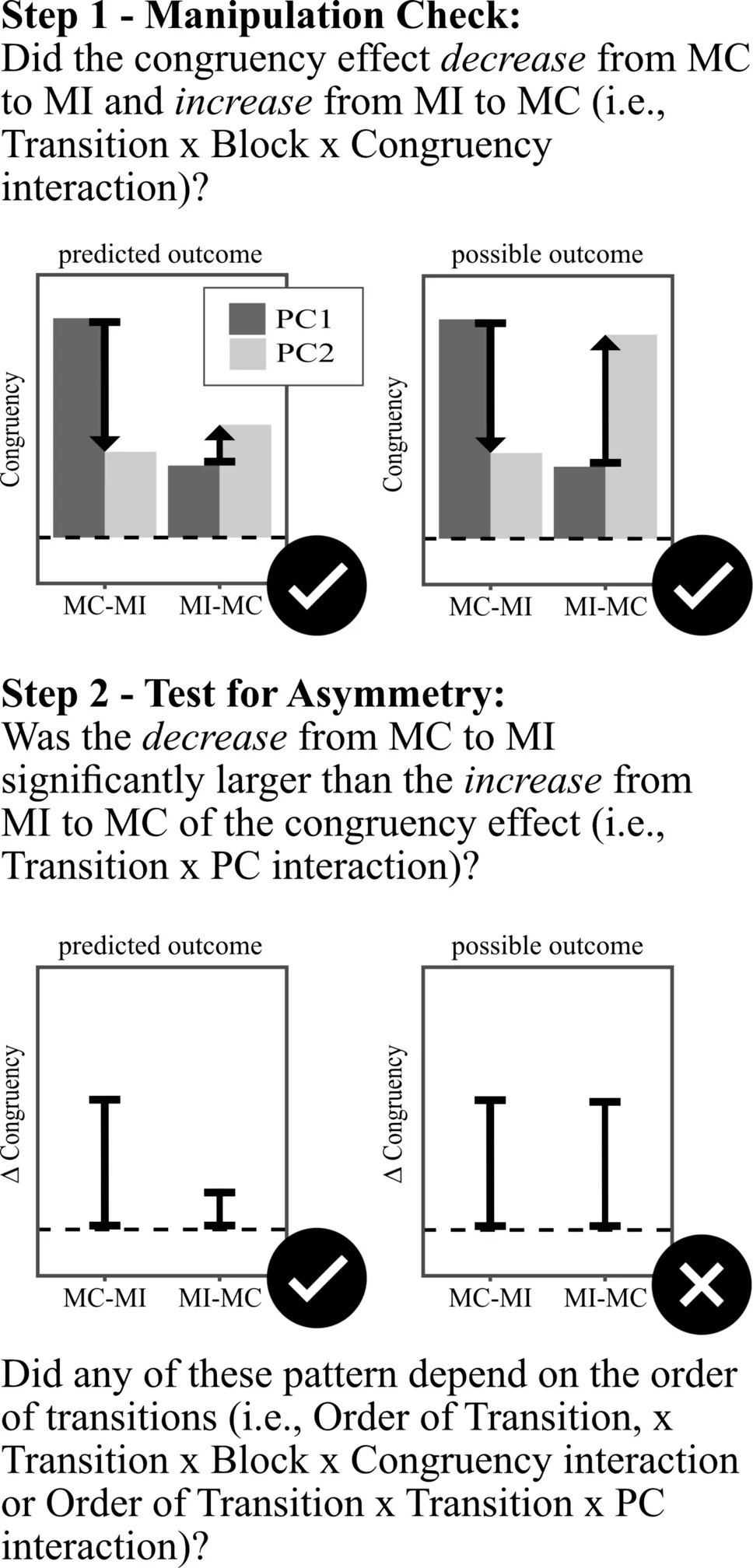
Schematic representation of the two-step data analysis procedure. The figure shows predicted and possible outcomes illustrating the need for the two-step analysis. Note that the first-step analysis would indicate a significant interaction between Transition, Block, and Congruency for any sufficiently strong decrease in the MC-MI transition paired with any increase in the MI-MC transition, regardless of the magnitude of the changes in the congruency effect. Thus, it does not test for an asymmetry in the change of the congruency effect. The second-step analysis would directly test for the asymmetry and indicate a significant interaction between Transition and PC on Congruency, for example, if the decrease from MC to MI in the MC-MI transition exceeds the increase from MC to MI in the MI-MC transition. Note that ∆ Congruency represents the absolute difference between PC1 and PC2

To investigate the ALS effect, we followed Abrahamse et al. (2013) and analyzed the changes in the magnitude of the congruency effect between the MC-MI and MI-MC transitions in a two-step procedure. The first step served as a manipulation check confirming opposite changes in congruency effects across the two transitions (i.e., decreasing during the MC-MI transition and increasing during the MI-MC transition), and the second step tested the asymmetry of this adaptation (see Fig. 1 for a detailed illustration of the rationale).

Specifically, in the first step, we employed a 2 (Order of Transition: MC-MI first, MI-MC first) × 2 (Transition: MC-MI, MI-MC) × 2 (Block: PC1, PC2) × 2 (Congruency: congruent, incongruent) mixed-factorial design with RTs and ERRs as dependent variables. The Order of Transition served as a between-participants factor, while all other factors were manipulated within participants. This manipulation check tested whether the congruency effects changed in opposite directions across the two transitions, thereby producing the theoretically opposing dynamics of adaptation. We specifically tested for the three-way interaction between Transition, Block, and Congruency, showing a decrease of the congruency effect during the MC-MI transition and an increase during the MI-MC transition (for a schematic representation of the two-step data analysis procedure, see Fig. 1). Interactions involving the Order of Transition allow not only a comparison between the two order groups but – because the first-step analysis preserves the actual block sequence – also a comparison between the first transition (i.e., the MC-MI transition of the MC-MI first-order and the MI-MC transition of the MI-MC first-order) and the second transition (i.e., the MI-MC transition of the MC-MI first-order and the MC-MI transition of the MI-MC first-order), independent of transition type. This allows assessing whether performance depends on a transition’s temporal position within the experiment (e.g., practice or fatigue effects).

In the second step, we tested for the asymmetry of this adaptation, i.e., that the decrease in the congruency effect during the MC-MI transition (i.e., MC minus MI) should be significantly larger than the increase during the MI-MC transition (i.e., MC minus MI). Testing this requires directly comparing performance between MC and MI blocks within each transition. Because the factor Block (PC1, PC2) does not map onto MC and MI in the same way across transitions (PC1 corresponds to MC in the MC-MI transition but to MI in the MI-MC transition), we reorganized the data by PC condition and employed a 2 (Order of Transition: MC-MI first, MI-MC first) × 2 (Transition: MC-MI, MI-MC) × 2 (PC: MC, MI) mixed-factorial design with congruency effects as the dependent variable. Again, the Order of Transition served as a between-participants factor. The asymmetric adaptation is expected in a significant interaction between Transition and PC (for a schematic representation of the two-step data analysis procedure, see Fig. 1). The respective three-way interaction allows for a comparison of the ALS effect between both order groups and thus is informative about the extent to which prior experience with one type of transition influences adaptation during the subsequent, reversed transition.

### Results

#### Data preprocessing

The analyses were restricted to the test blocks, resulting in a dataset of 40,000 experimental trials (50 participants × 800 trials). The first trial of each block was excluded (0.50% of trials). Participants were excluded from analyses if their overall mean RT or ERR was more than three interquartile ranges above the third quartile or below the first quartile (see Tukey, 1977). Based on these criteria, data from 49 participants were included in the final analyses. The RT analysis was restricted to correct responses, excluding errors (4.60% of trials) and post-error trials (4.18% of trials). Additionally, extreme RTs that were smaller than 150 ms or larger than 3,000 ms (see J. Miller, 2023) were excluded (0.34% of trials).

An alpha level of .05 was used for all statistical tests, with partial eta-squared (ɳ_p_^2^) reported as the measure of effect size. All congruency effects were calculated as the difference between incongruent and congruent trials. Note that the focus is on effects that are of interest to the research questions. For archival purposes, the full ANOVA results for RTs and ERRs of the present and subsequent experiments are presented in Appendix A. Importantly, this procedure was applied consistently across all subsequent experiments.

#### Response time (RT) data

The 2 × 2 × 2 × 2 mixed-factors analysis of variance (ANOVA) on RTs included Order of Transition as a between-participants factor, along with Transition, Block, and Congruency as within-participants factors. The results showed a significant main effect of Congruency, *F*(1, 47) = 132.93, *p* <.001, ɳ_p_^2^ =.74, with longer RTs for incongruent trials (*M* = 801 ms ± *SD* = 189) compared to congruent trials (*M* = 710 ms ± *SD* = 148). This congruency effect was further modulated by a significant interaction with Transition and Block, *F*(1, 47) = 50.86, *p* <.001, ɳ_p_^2^ =.52.^4^ The post hoc analysis indicated a substantial decrease in the congruency effect in the MC-MI transition, *F*(1, 47) = 55.69, *p* <.001, ɳ_p_^2^ =.54 (mean congruency effects of 158 ms and 43 ms for PC1 and PC2 blocks, respectively), in contrast to the increase in the congruency effect in the MI-MC transition, *F*(1, 47) = 22.93, *p* <.001, ɳ_p_^2^ =.33 (mean congruency effects of 43 ms and 118 ms for PC1 and PC2 blocks, respectively). The general RT decrease between sessions (i.e., between the first and the second transition) is reflected in the significant interaction between Order of Transition and Transition, *F*(1, 47) = 129.87, *p* <.001, ɳ_p_^2^ =.73. To illustrate, the significant interaction with the order of transition indicates that the effect of Transition depended on its actual *position* within the experimental sequence. While a main effect of Transition would suggest that one transition type (MC-MI or MI-MC) generally led to longer RTs, the interaction with the order of transition shows that RTs were longer for the *first* transition – regardless of type – than for the *second*. Likewise, the general decrease of congruency effects between sessions (i.e., between the first and the second transition) is reflected in the significant interaction between Order of Transition, Transition, and Congruency, *F*(1, 47) = 21.13, *p* <.001, ɳ_p_^2^ =.31.

**Fig. 2:**
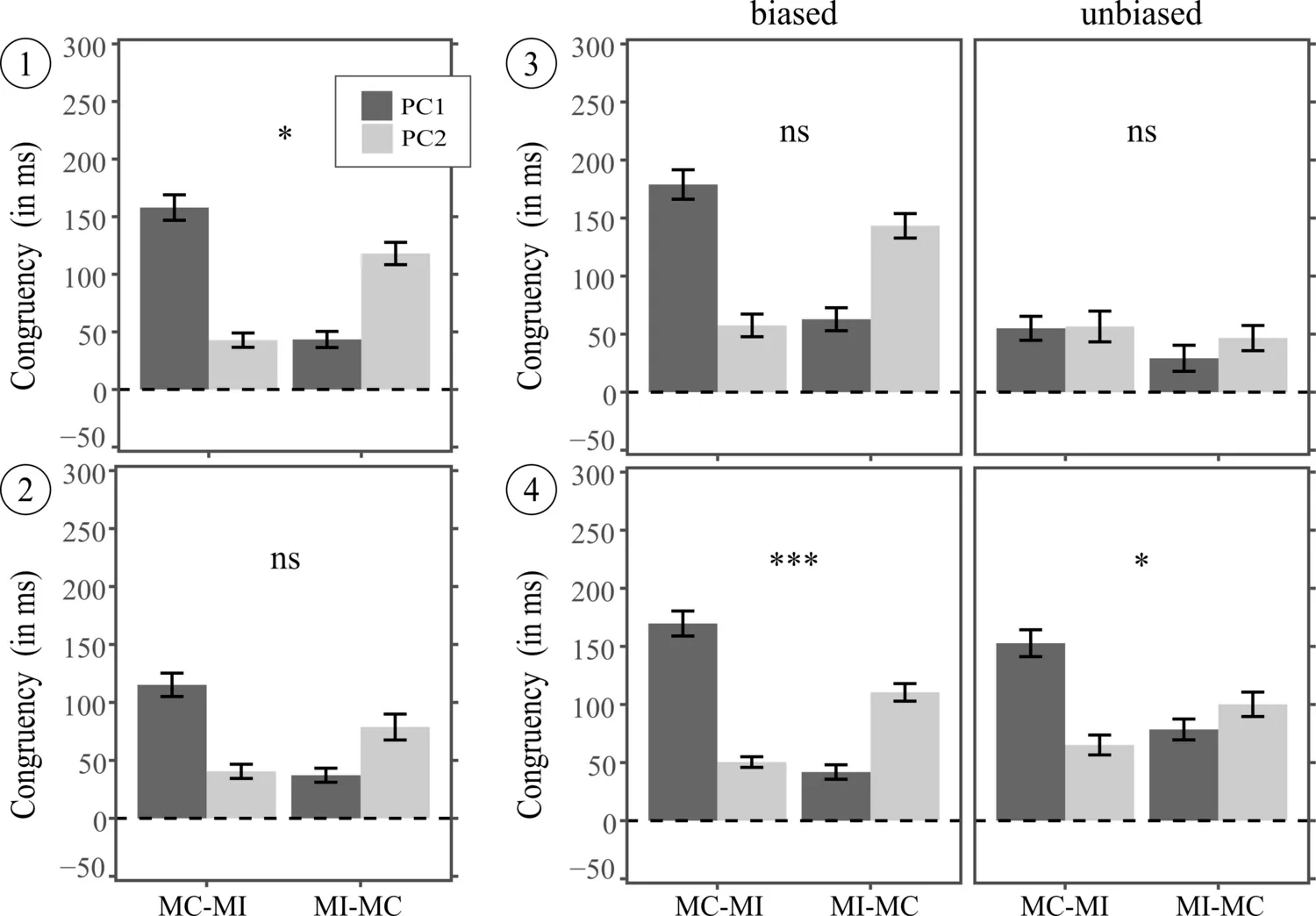
Mean congruency effects across experiments presented as a function of transition, block, and (for Experiments 3 and 4) item type. The number within each circle indicates the corresponding experiment. The indicated significance in the graphs refers to observing an asymmetric list shift (ALS) effect (i.e., a larger congruency decrease in the MC-MI transition than a congruency increase in the MI-MC transition). The error bars represent the standard errors of the mean; MC = mostly congruent; MI = mostly incongruent; ns = not significant; * *p* <.05; *** *p* <.001

**Fig. 3:**
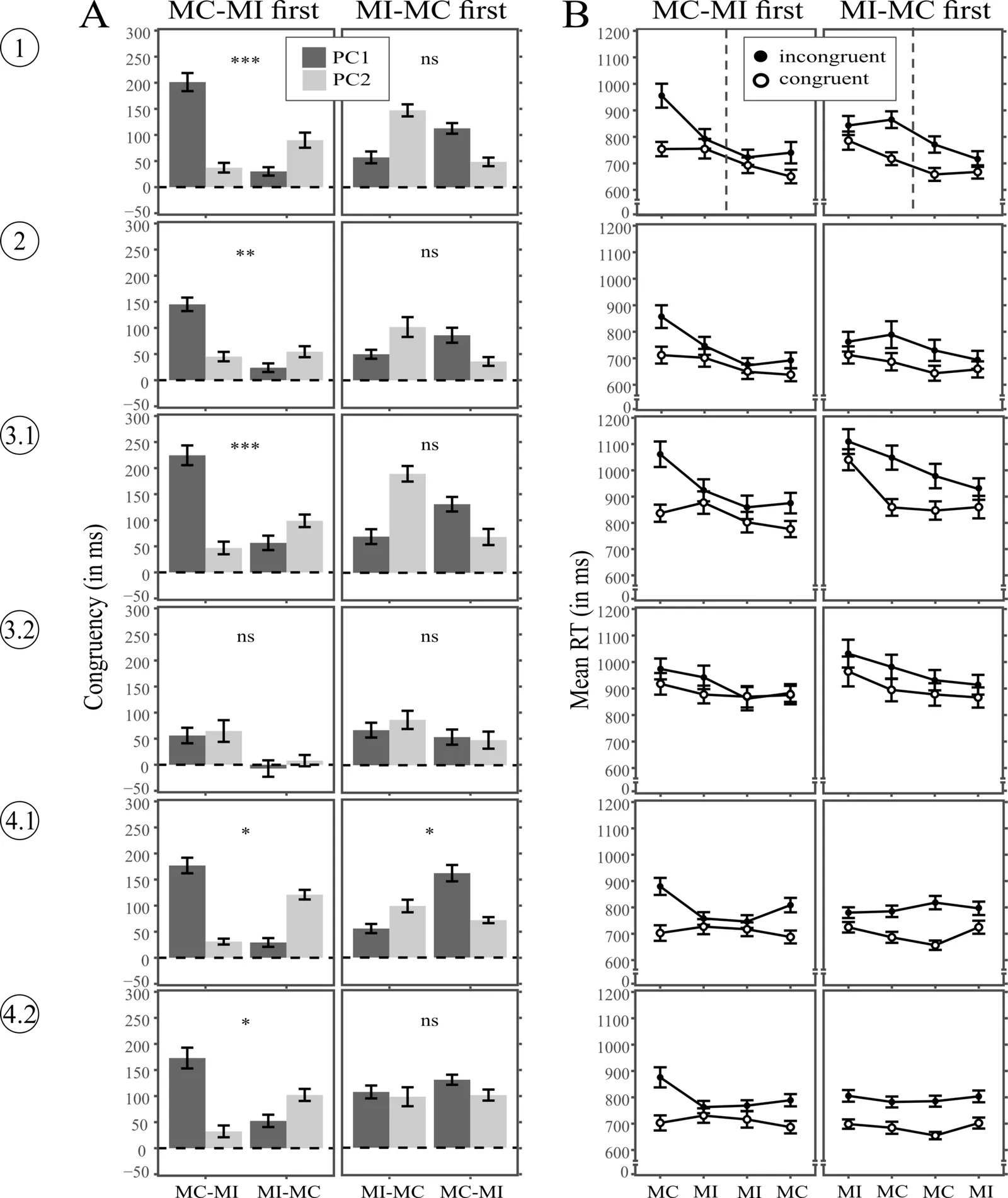
Mean congruency effects on response time (**A**) across experiments presented as a function of order of transition, transition, block, and (for Experiments 3 and 4) item type and mean response times (**B**) across experiments presented as a function of order of transition, block sequence (i.e., the temporal order of the blocks), congruency, and for Experiments 3 and 4, item type. The number within each circle corresponds to the relevant experiment, with Experiments 3 and 4 subdivided into 3.1/4.1 for biased items and 3.2/4.2 for unbiased items. The indicated significance in the graphs refers to observing an asymmetric list shift (ALS) effect (i.e., a larger congruency decrease in the MC-MI transition than a congruency increase in the MI-MC transition). The error bars represent the standard errors of the mean. The dashed vertical line (1B) marks the delay between sessions; MC = mostly congruent; MI = mostly incongruent; ns = not significant; * *p* <.05; ** *p* <.01; *** *p* <.001

To test for the asymmetry of the adaptation, we conducted a 2 × 2 × 2 mixed-factors ANOVA on congruency effects, using Order of Transition as a between-participants factor and Transition and PC as within-participants factors. Most importantly for the hypothesis, an ALS effect was observed, as indicated by the significant interaction between Transition and PC, *F*(1, 47) = 6.03, *p* =.018, ɳ_p_^2^ =.11: The decrease in the congruency effect in the MC-MI transition (115 ms; MC minus MI) was larger than the increase observed in the MI-MC transition (75 ms; MC minus MI; see Fig. 2, Sect. 1). Furthermore, the factor Order of Transition allowed a comparison of the ALS effects (i.e., the interaction between Transition and PC) between the MC-MI first and the MI-MC first-order groups, revealing the extent to which prior experience with one type of transition influences adaptation during the subsequent, reversed transition. Indeed, the corresponding three-way interaction was significant, *F*(1, 47) = 16.61, *p* <.001, ɳ_p_^2^ =.26. For participants in the MC-MI first-order group, the interaction between Transition and PC was significant, *F*(1, 24) = 25.65, *p* <.001, ɳ_p_^2^ =.52, demonstrating an ALS effect: The decrease in the congruency effect in the MC-MI transition (164 ms; MC minus MI) was larger than the increase observed in the MI-MC transition (60 ms; MC minus MI). Conversely, in the MI-MC first-order group, the interaction between Transition and PC was not significant, *F*(1, 23) = 1.11, *p* =.303, ɳ_p_^2^ =.05, indicating no meaningful ALS effect (see Fig. 3, Sect. 1A).

#### Error data

The overall ERR was 5.25%. The corresponding 2 × 2 × 2 × 2 mixed-factors ANOVA on ERRs showed a significant main effect of Congruency, *F*(1, 47) = 13.09, *p* <.001, ɳ_p_^2^ =.22, with more errors occurring in incongruent trials (*M* = 6.03% ± *SD* = 5.23) compared to congruent trials (*M* = 4.47% ± *SD* = 3.76). This congruency effect was further modulated by a significant interaction with Transition and Block, *F*(1, 47) = 25.75, *p* <.001, ɳ_p_^2^ =.35. Post hoc analyses indicated a substantial reduction in the congruency effect in the MC-MI transition, *F*(1, 47) = 21.51, *p* <.001, ɳ_p_^2^ =.31 (mean congruency effects of 3.91% and −0.57% for PC1 and PC2 blocks, respectively), compared to an increase in the congruency effect in the MI-MC transition, *F*(1, 47) = 6.41, *p* =.015, ɳ_p_^2^ =.12 (mean congruency effects of 0.18% and 2.73% for PC1 and PC2 blocks, respectively).

However, the subsequent 2 × 2 × 2 mixed-factors ANOVA on congruency effects revealed no evidence for an ALS effect, *F*(1, 47) = 1.81, *p* =.185, ɳ_p_^2^ =.04. The congruency effect in the MC-MI transition (4.48%; MC minus MI) was only descriptively larger than the increase in the MI-MC transition (2.55%; MC minus MI). This result was not affected by the Order of Transition, *F* < 1. No ALS effects were observed for either the MC-MI first-order group, *F*(1, 24) = 2.90, *p* =.102, ɳ_p_^2^ =.11, or the MI-MC first-order group, *F* < 1.

### Discussion

In Experiment 1, we aimed to determine whether the ALS effect initially observed by Abrahamse et al. (2013) in a between-participants design would also be evident in a within-participants design. To this end, all participants performed both transitions from a PC1 block (MC or MI) to a PC2 block (MI or MC) in two separate sessions. Consistent with Abrahamse et al. (2013), our design revealed an overall ALS effect, extending previous research by demonstrating it in a within-participants design: The decrease in the congruency effect during the MC-MI transition was significantly larger than the increase during the MI-MC transition. To our knowledge, this is the first demonstration of an ALS effect using a full within-participants design.

However, further analysis revealed that the overall pattern of the ALS effect originated exclusively from the MC-MI first-order and was entirely absent in the group with the reversed transition order (i.e., MI-MC first-order). Additionally, visual inspection of mean RTs over time suggests that performance on a given trial type^5^ improves (i.e., RTs become shorter) when transitioning from a block where a trial type is rare to one where it is more frequent. In the MC-MI first-order, for example, large RTs for rare incongruent trials in MC blocks of the first session substantially decrease in the subsequent MI block when incongruent trials are frequent (see Fig. 3, Sect. 1B). Moreover, across both order groups, the results provide clear evidence of practice effects in the within-participants design. Performance improves substantially from the first to the second session (see Fig. 3, Sect. 1B, dashed vertical line for visualization).

Importantly, the combination of different practice effects in an LWPC experiment (i.e., trial-type practice combined with a general RT decrease) could potentially mimic the ALS effect, as long as it is also assumed that congruency effects diminish with practice (Schmidt, 2016). More specifically, during the MC-MI transition, both the increased frequency of incongruent trials and the general decrease in congruency effects observed with practice predict a reduction of the congruency effect. Conversely, for the opposite MI-MC transition, the shift to more frequent congruent trials predicts larger congruency effects, which, however, is counteracted by the general decrease in congruency effects observed with practice, potentially reducing the congruency effect (see Schmidt, 2016). Additionally, the substantial performance improvements from the first to the second transition – evidenced by a general decrease in RTs and likely accompanied by practice benefits^6^ – may support the emergence of an ALS pattern in the MC-MI first-order group. However, these same practice-related benefits could diminish or obscure the effect in the MI-MC first-order group, potentially working against the detection of ALS in that direction.

In summary, while the ALS effect was significant for RTs, the data also suggest that asymmetric effects depend on the order of transition and that a combination of different practice effects may potentially account for the observed pattern (see also Schmidt, 2016).

## Experiment 2

In Experiment 2, we aimed to address the potential influence of practice on the ALS effect. To achieve this, we conducted the entire experiment in a single session and included additional frequency-unbiased blocks (50% congruent and 50% incongruent trials) prior to each transition. The purpose of these baseline blocks was twofold: First, to ensure equal practice and exposure to all color-word combinations for both trial types, and second, to reduce the general decline in RT that occurred throughout the experiment. By providing substantial practice before the critical transition phase, these additional frequency-unbiased trials help participants’ performance stabilize early, preventing the pronounced practice-related RT decline that would otherwise occur later in the experiment. The blocks also allowed us to more directly test the extent to which the congruency effects depend on the overall RT level and practice.

### Method

#### Participants

Building on Experiment 1, Experiment 2 included 50 participants (43 females; *M*_*age*_ = 20.76 years, *SD* = 4.13), 96% of whom were students. Participants were recruited through the participant pool management software Sona Systems from the Psychology Department at the University of Regensburg and received partial course credit for their participation. Data collection took place between November 2, 2023, and February 12, 2024. All participants were native German speakers with normal color vision.

#### Apparatus and stimuli

The apparatus and stimuli were the same as in Experiment 1, except that the color-word Stroop task was programmed using a more recent version of PsychoPy (v2023.2.2; Peirce et al., 2019, 2022).

#### Procedure

Experiment 2 was a conceptual replication of Experiment 1 with two major modifications. First, instead of conducting two sessions, both transitions (MC-MI, MI-MC) were completed in a single session. As in Experiment 1, the order of transitions was counterbalanced across participants. Second, to reduce potential practice effects for frequently occurring trial types, each transition was preceded by a frequency-unbiased block of 200 trials, consisting of 50% congruent (100 trials, 25 per color) and 50% incongruent (100 trials, 25 per color) trials. Thus, the experimental sequence consisted of a frequency-unbiased block, followed by the first transition (MC-MI or MI-MC), followed by another frequency-unbiased block, and finally the second transition (MI-MC or MC-MI). These frequency-unbiased blocks ensured balanced practice exposure across all paired color-word combinations. The specific proportions of congruent and incongruent trials in the frequency-unbiased blocks are summarized in Table 1. Each participant completed both transitions (MC-MI and MI-MC) in four experimental blocks of 200 trials each, along with two frequency-unbiased blocks of 200 trials, each preceding a transition, totaling 1,200 trials per participant. The entire procedure took approximately 35 min, during which RTs and ERRs were recorded.

#### Design

To investigate the ALS effect, we again followed the same two-step procedure as in Experiment 1.

### Results

#### Data preprocessing

The same outlier procedure used in Experiment 1 was also applied in Experiment 2. Because we aimed to analyze the frequency-unbiased blocks separately in an exploratory analysis, we preprocessed the entire dataset, resulting in a total of 60,000 trials (50 participants × 1,200 trials). The first trial of each block was excluded (0.50% of trials). Based on the outlier criteria, data from 49 participants were included in the final analyses (see Tukey, 1977). The RT analysis was restricted to correct responses, excluding errors (4.96% of trials) and post-error trials (4.44% of trials). Additionally, extreme RTs were excluded (0.78% of trials; see J. Miller, 2023). RT and ERR analyses were restricted to the test blocks.

#### RT data

Similar to Experiment 1, the 2 × 2 × 2 × 2 mixed-factors ANOVA on RTs, with Order of Transition as a between-participants factor and Transition, Block, and Congruency as within-participants factors, revealed a significant main effect of Congruency, *F*(1, 47) = 72.66, *p* <.001, ɳ_p_^2^ =.61, with longer RTs for incongruent trials (*M* = 743 ms ± *SD* = 192) compared to congruent trials (*M* = 675 ms ± *SD* = 150). This congruency effect was further modulated by a significant interaction with Transition and Block, *F*(1, 47) = 21.93, *p* <.001, ɳ_p_^2^ =.32, denoting an overall LWPC effect. The post hoc analysis indicated a substantial decrease in the congruency effect in the MC-MI transition, *F*(1, 47) = 30.08, *p* <.001, ɳ_p_^2^ =.39 (mean congruency effects of 115 ms and 41 ms for PC1 and PC2 blocks, respectively), in contrast to the increase in the congruency effect in the MI-MC transition, *F*(1, 47) = 5.96, *p* =.018, ɳ_p_^2^ =.11 (mean congruency effects of 37 ms and 79 ms for PC1 and PC2 blocks, respectively). As in Experiment 1, the interaction between Order of Transition and Transition shows that participants generally responded faster in the second transition than in the first, *F*(1, 47) = 24.02, *p* <.001, ɳ_p_^2^ =.34. Furthermore, the general decrease of congruency effects between the first and the second transition is reflected in the significant interaction between Order of Transition, Transition, and Congruency, *F*(1, 47) = 13.17, *p* <.001, ɳ_p_^2^ =.22.

However, the subsequent 2 × 2 × 2 mixed-factors ANOVA on congruency effects using Order of Transition as a between-participants factor and Transition and PC as within-participants factors showed that the decrease in the congruency effect in the MC-MI transition (74 ms; MC minus MI) was only descriptively larger than the increase in the MI-MC transition (42 ms; MC minus MI), *F*(1, 47) = 3.45, *p* =.070, ɳ_p_^2^ =.07. This suggests that there is no statistically significant ALS effect, although the descriptive pattern of results resembles those of Experiment 1 (see Fig. 2, Sect. 2). Furthermore, the three-way interaction between Order of Transition, Transition, and PC just missed the level of statistical significance, *F*(1, 47) = 3.85, *p* =.056, ɳ_p_^2^ =.08. For participants in the MC-MI first-order group, the interaction between Transition and PC was significant, *F*(1, 23) = 9.03, *p* =.006, ɳ_p_^2^ =.28, revealing an ALS effect: The decrease in the congruency effect in the MC-MI transition (100 ms; MC minus MI) was larger than the increase observed in the MI-MC transition (31 ms; MC minus MI). In contrast, in the MI-MC first-order group, the interaction between Transition and PC was not significant, *F* < 1, indicating no meaningful ALS effect (see Fig. 3, Sect. 2A).

#### Error data

The overall ERR was 5.75%, similar to that of Experiment 1. The corresponding 2 × 2 × 2 × 2 mixed-factors ANOVA on ERRs revealed a significant main effect of Congruency, *F*(1, 47) = 5.84, *p* =.020, ɳ_p_^2^ =.11, with more errors occurring in incongruent trials (*M* = 6.29% ± *SD* = 4.95) compared to congruent trials (*M* = 5.21% ± *SD* = 4.13). This congruency effect was further modulated by a significant interaction with Transition and Block, *F*(1, 47) = 39.82, *p* <.001, ɳ_p_^2^ =.46. Post hoc analyses indicated a pronounced reduction in the congruency effect in the MC-MI transition, *F*(1, 47) = 13.39, *p* <.001, ɳ_p_^2^ =.22 (mean congruency effects of 3.36% and −0.34% for PC1 and PC2 blocks, respectively), compared to an increase in the congruency effect in the MI-MC transition, *F*(1, 47) = 11.81, *p* =.001, ɳ_p_^2^ =.20 (mean congruency effects of −0.97% and 2.26% for PC1 and PC2 blocks, respectively).

However, the subsequent 2 × 2 × 2 mixed-factors ANOVA on congruency effects revealed no evidence for an ALS effect, *F* < 1, as there was no significantly larger decrease in the congruency effect in the MC-MI transition (3.70%; MC minus MI) than the increase in the MI-MC transition (3.23%; MC minus MI). This result was not affected by the Order of Transition, *F* < 1. No ALS effects were observed for either the MC-MI first-order group, *F* < 1, or the MI-MC first-order group, *F* < 1.

#### Additional analyses

To test whether nonspecific general practice affects the present congruency effect and may thus contribute to ALS effects (see Schmidt, 2016), we compared the congruency effect between the first and second frequency-unbiased blocks. We conducted a 2 (Order of Transition: MC-MI first, MI-MC first) × 2 (Position: first, second) × 2 (Congruency: congruent, incongruent) mixed-factorial design. The Order of Transition served as a between-participants factor, while all other factors were manipulated within participants. First of all, there was a strong effect of nonspecific, general practice, as indicated by the main effect of Position for RTs, *F*(1, 47) = 55.94, *p* <.001, ɳ_p_^2^ =.54. Despite this general RT decrease, there was only a descriptive but not significant reduction in the congruency effect over time within the frequency-unbiased blocks for RTs and ERRs, as reflected by a non-significant interaction between Position and Congruency (both *p* ≥.173). Additional analyses examining the congruency effect over time for each Order of Transition also showed no significant reduction for either RTs or ERRs (all *F*s ≤ 2.87, all *p*s ≥.104, all ɳ_p_^2^s ≤.11).

### Discussion

Experiment 2 was conducted as a single-session study, with each transition preceded by a frequency-unbiased baseline block consisting of 50% congruent and 50% incongruent trials. The result pattern closely mirrored that of Experiment 1 (see Fig. 3, compare Sects. 1 and 2). Although the modulation of the ALS effect by the order of transition narrowly missed statistical significance, asymmetric list shifting was observed only for the MC-MI first-order, not for the MI-MC first-order. While the frequency-unbiased baseline blocks ensured ample trial-type practice prior to each transition, they did not prevent further nonspecific, general practice throughout the experiment. That is, participants generally responded faster in the later frequency-unbiased baseline block of Experiment 2, indicating clear improvements over time. Although we did not find evidence that the congruency effect was largely affected by general practice in the frequency-unbiased blocks, congruency effects nevertheless declined across the two transitions.

Taken together, Experiment 2 did not succeed in reducing the general RT decrease, as both RTs were faster and the congruency effect was smaller in the second transition than in the first, providing clear evidence for practice effects. At this point, we cannot rule out that different types of practice mimic the pattern of the ALS effect.

## Experiment 3

So far, we can argue that the ALS effect can also be observed in a within-participants design; however, it appears to depend on a specific order of transition, as it was only observed in the group that started with the MC-MI transition. Both previous experiments included different levels of trial-type practice in the PC conditions and were subject to substantial nonspecific practice (which was not prevented in Experiment 2, despite the use of frequency-unbiased blocks prior to each transition). This may account for the differences in the ALS effect across the two order groups. Therefore, in Experiment 3, we incorporated a set of frequency-unbiased *items* (50% congruent and 50% incongruent) into the MC and MI blocks (Bugg et al., 2011). Unlike biased items, these frequency-unbiased items balanced stimulus-specific practice across conditions (i.e., each stimulus was equally likely to be congruent or incongruent in all blocks), while trial-type practice persisted through their embedding within the PC-manipulated MC and MI blocks. Consequently, a potential ALS effect in those conditions could not be attributed to differential stimulus-specific practice in the MC and MI blocks.

### Method

#### Participants

Similar to the previous experiments, Experiment 3 included 50 participants (34 females; *M*_*age*_ = 26.44 years, *SD* = 9.20), 80% of whom were students. Participants were recruited through the participant pool management software, Sona Systems, from the Department of Psychology at the University of Regensburg. They received either partial course credit or €4 for their participation. Data collection took place between April 23, 2024, and June 21, 2024. All participants were native German speakers with normal color vision.

#### Apparatus and stimuli

The apparatus was identical to Experiment 2. To investigate the potential transferability of the ALS effect to a set of frequency-unbiased items, we slightly modified the stimuli used in Experiment 3. The Stroop stimuli consisted of six German color words (in English: *RED*, *BLUE*, *YELLOW*, *GREEN*, *PURPLE*, *PINK*) presented in the corresponding print colors from PsychoPy’s standard palette (“red,” “blue,” “yellow,” “green,” “purple,” and “magenta”). The words were presented in Arial font (letter height 0.05), centered on the screen against a gray (“gray”) background. The task followed a 6-AFC paradigm, divided into three alternating subsets with non-overlapping stimulus and response sets (subset 1: RED and GREEN; subset 2: BLUE and YELLOW; subset 3: PURPLE and PINK; e.g., the word RED never appeared in blue; see Blais & Bunge, 2010; Bugg et al., 2008; Schmidt, 2014). This design ensured that each color word was presented either in its respective congruent print color (e.g., RED in red) or in a predetermined incongruent print color (e.g., RED in green), resulting in a total of 12 unique stimuli drawn from the full 6 × 6 stimulus matrix. Participants responded to the stimuli using color-specific keys on the keyboard. Responses were made with the ring (S, L), middle (D, K), and index (F, J) fingers of both hands, with each subset of stimuli uniquely assigned to homologous finger pairs of the left and right hands (e.g., red was mapped to the left middle finger and green to the right middle finger). Responses to purple, red, and blue were always made with the left hand, while responses to yellow, green, and pink were consistently made with the right hand. Stimulus subsets were assigned to homologous finger pairs in a counterbalanced manner across participants, resulting in three distinct stimulus–response (S-R) mappings. In the first mapping, the keys S, D, F, J, K, and L corresponded to the colors purple, red, blue, yellow, green, and pink, respectively. In the second mapping, the same keys matched blue, purple, red, green, pink, and yellow. In the third mapping, the keys corresponded to red, blue, purple, pink, yellow, and green.

#### Procedure

As in Experiment 2, Experiment 3 was conducted in a single session. The experiment started with the same two blocks of practice trials as Experiments 1 and 2, but with two more colors added. This resulted in 36 practice trials (six trials per color), where participants responded to colored squares, followed by 72 Stroop task trials (six trials for each color-word combination). After practice, all participants completed both transitions from a PC1 block (MC or MI) to a PC2 block (MI or MC). To separate the two transitions (counterbalanced across participants), a 2-min distractor task was implemented between transitions. Specifically, participants had to complete a dot detection task in which they needed to press the space bar whenever a white circle (i.e., the letter “o” written in “white”, letter height 0.05) appeared at a predetermined location on the right, center, or left of the screen every 0–2 s at a random time.

The color-word combinations from subsets 1 and 2 served as biased items, with 80% congruent items in the MC blocks and 80% incongruent items in the MI blocks. The unbiased items, which comprised the color-word combinations from subset 3, were presented equally in their congruent (18 trials, nine per color) and in predetermined incongruent print colors (18 trials, nine per color), resulting in a list-wide PC of 50% (i.e., PC-50). The overall ratio of unbiased to biased items in the test blocks was determined based on the percentage structure outlined by Bugg et al. (2011). Therefore, an MC block that included both biased (PC-80) and unbiased (PC-50) items comprised 75% congruent trials (160 PC-80 plus 18 PC-50 items) and 25% incongruent trials (40 PC-80 plus 18 PC-50 items). Conversely, an MI block containing both biased (PC-20) and unbiased (PC-50) items consisted of 25% congruent trials (40 PC-20 plus 18 PC-50 items) and 75% incongruent trials (160 PC-20 plus 18 PC-50 items). The order of stimulus presentation within each block was pseudorandomized to prevent direct repetition of subsets in consecutive trials (e.g., left index finger is never followed by right index finger). This design ensured that stimuli were not repeated and required participants to alternate their responding homologous finger pair on each trial. The specific proportions of congruent and incongruent trials in the MC and MI blocks are summarized in Table 1.

Each participant completed both transitions (MC-MI and MI-MC) in four blocks of 236 trials each, totaling 944 trials per participant. The entire experiment took approximately 30 min, during which RTs and ERRs were recorded. All other aspects of the procedure were the same as those in Experiment 1.

#### Design

The design was identical to that of Experiment 1, except that we applied the two-step procedure separately for each item type (biased, unbiased), following the approach used in previous studies (e.g., Bugg, 2014; Bugg et al., 2011).

### Results

#### Data preprocessing

The same outlier procedure used in Experiment 1 was applied in Experiment 3. All analyses were restricted to test blocks, resulting in a dataset of 47,200 experimental trials (50 participants × 944 trials). The first trial of each block was excluded (0.42% of trials). Based on the outlier criteria, data from 49 participants were included in the final analyses (see Tukey, 1977). The RT analysis was restricted to correct responses, excluding errors (7.92% of trials) and post-error trials (6.43% of trials). Additionally, extreme RTs were excluded (1.62% of trials; see J. Miller, 2023).

#### Biased items (PC-80 and PC-20 items)

##### RT data

The 2 × 2 × 2 × 2 mixed-factors ANOVA on RTs of biased items with Order of Transition as a between-participants factor and Transition, Block, and Congruency as within-participants factors revealed a significant main effect of Congruency, *F*(1, 47) = 133.25, *p* <.001, ɳ_p_^2^ =.74, with longer RTs for incongruent trials (*M* = 972 ms ± *SD* = 233) compared to congruent trials (*M* = 862 ms ± *SD* = 195). This congruency effect was further modulated by a significant interaction with Transition and Block, *F*(1, 47) = 51.30, *p* <.001, ɳ_p_^2^ =.52, indicating an overall LWPC effect. The post hoc analysis indicated a substantial decrease in the congruency effect in the MC-MI transition, *F*(1, 47) = 35.70, *p* <.001, ɳ_p_^2^ =.43 (mean congruency effects of 179 ms and 58 ms for PC1 and PC2 blocks, respectively), in contrast to the increase in the congruency effect in the MI-MC transition, *F*(1, 47) = 28.97, *p* <.001, ɳ_p_^2^ =.38 (mean congruency effects of 63 ms and 143 ms for PC1 and PC2 blocks, respectively). There was again a significant interaction between Order of Transition and Transition, indicating that participants generally responded faster in the second transition than in the first, *F*(1, 47) = 63.29, *p* <.001, ɳ_p_^2^ =.57. Again, the general decrease of congruency effects between the first and the second transition is reflected in the significant interaction between Order of Transition, Transition, and Congruency, *F*(1, 47) = 10.48, *p* =.002, ɳ_p_^2^ =.18.

However, the subsequent 2 × 2 × 2 mixed-factors ANOVA on congruency effects using Order of Transition as a between-participants factor and Transition and PC as within-participants factors suggests that the decrease in the congruency effect in the MC-MI transition (121 ms; MC minus MI) was only descriptively larger than the increase in the MI-MC transition (80 ms; MC minus MI), *F*(1, 47) = 3.19, *p* =.080, ɳ_p_^2^ =.06. This suggests that there is no statistically significant ALS effect, although the descriptive pattern of results is like that observed in Experiment 1 (see Fig. 2, Sect. 3, left panel). The interaction between the Order of Transition, Transition, and PC, *F*(1, 47) = 19.61, *p* <.001, ɳ_p_^2^ =.29, however, again shows that the ALS effect strongly depends on the order of the transitions. For participants in the MC-MI first-order group, the interaction between Transition and PC was significant, *F*(1, 24) = 19.06, *p* <.001, ɳ_p_^2^ =.44, revealing an ALS effect: The decrease in the congruency effect in the MC-MI transition (178 ms; MC minus MI) was larger than the increase observed in the MI-MC transition (42 ms; MC minus MI). In contrast, in the MI-MC first-order group, the interaction between Transition and PC was not significant, *F*(1, 23) = 3.55, *p* =.072, ɳ_p_^2^ =.13, indicating no meaningful ALS effect (see Fig. 3, Sect. 3.1A).

##### Error data

The overall ERR for biased items was 8.17%. The corresponding 2 × 2 × 2 × 2 mixed-factors ANOVA on ERRs of biased items revealed a significant main effect of Congruency, *F*(1, 47) = 11.31, *p* =.002, ɳ_p_^2^ =.19, with more errors occurring in incongruent trials (*M* = 8.84% ± *SD* = 8.56) compared to congruent trials (*M* = 7.50% ± *SD* = 8.57). However, there was no significant three-way interaction of Transition, Block, and Congruency, *F*(1, 47) = 1.86, *p* =.180, ɳ_p_^2^ =.04.

The subsequent 2 × 2 × 2 mixed-factors ANOVA on congruency effects revealed no evidence for an ALS effect, *F*(1, 47) = 2.53, *p* =.118, ɳ_p_^2^ =.05, which was not affected by the Order of Transition, *F*(1, 47) = 2.04, *p* =.160, ɳ_p_^2^ =.04. No ALS effects were observed for either the MC-MI first-order group, *F*(1, 24) = 3.68, *p* =.067, ɳ_p_^2^ =.13, or the MI-MC first-order group, *F* < 1.

#### Unbiased items (PC-50 items)

##### RT data

The analogous 2 × 2 × 2 × 2 mixed-factors ANOVA on RTs of unbiased items with Order of Transition as a between-participants factor and Transition, Block, and Congruency as within-participants factors showed a significant main effect of Congruency, *F*(1, 47) = 31.96, *p* <.001, ɳ_p_^2^ =.41, with longer RTs for incongruent trials (*M* = 939 ms ± *SD* = 212) compared to congruent trials (*M* = 893 ms ± *SD* = 204). However, the three-way interaction of Transition, Block, and Congruency did not reach significance, *F* < 1, indicating the absence of an overall LWPC effect. The interaction between Order of Transition and Transition confirms that even for unbiased items, participants generally responded faster in the second transition than in the first, *F*(1, 47) = 16.81, *p* <.001, ɳ_p_^2^ =.26. Additionally, the general decrease of congruency effects between the first and the second transition is reflected in the significant interaction between Order of Transition, Transition, and Congruency, *F*(1, 47) = 7.78, *p* =.008, ɳ_p_^2^ =.14.

The subsequent 2 × 2 × 2 mixed-factors ANOVA on congruency effects using Order of Transition as a between-participants factor and Transition and PC as within-participants factors revealed no evidence for an ALS effect for unbiased items, *F* < 1 (see Fig. 2, Sect. 3, right panel), which was not affected by the Order of Transition, *F* < 1. No ALS effects were observed for either the MC-MI first-order group, *F* < 1, or the MI-MC first-order group, *F* < 1 (see Fig. 3, Sect. 3.2A).

##### Error data

The overall ERR for unbiased items was 8.86%. The corresponding 2 × 2 × 2 × 2 mixed-factors ANOVA on ERRs of unbiased items showed no significant effects, all *F*s ≤ 3.51, all *p*s ≥.067, all ɳ_p_^2^s ≤.07.

The subsequent 2 × 2 × 2 mixed-factors ANOVA on congruency effects also revealed no evidence for an ALS effect, *F* < 1, which was not affected by the Order of Transition, *F* < 1. No ALS effects were observed for either the MC-MI first-order group, *F* < 1, or the MI-MC first-order group, *F* < 1.

### Discussion

We conducted Experiment 3 to further investigate whether an ALS effect can be observed within participants when stimulus-specific practice is controlled for. To this end, Experiment 3 included a different set of frequency-unbiased (i.e., 50% congruent) items embedded in the MC and MI blocks, which should not be affected by selective practice of frequent stimulus types. For the biased item set, the results replicated the findings of Experiment 2: A strong, significant ALS effect was again only found for the MC-MI first-order group, but not for the MI-MC first-order group. Critically, unbiased items that were not subjected to stimulus-specific practice showed neither an ALS effect nor an LWPC effect (as evidenced by the non-significant interaction between Transition, Block, and Congruency). Instead, their RTs decreased rather consistently for both congruent and incongruent trials over time, indicating that performance changes for biased items also result from stimulus-specific learning rather than trial-type practice adaptation alone. Additionally, a general decrease in RT and a concurrent reduction in congruency effects were observed over the course of the experiment for both item sets.

Taken together, these findings suggest that the combination of different types of practice may possibly account for the differences in the ALS effect in both order groups: It may support the emergence of an ALS pattern in the MC-MI first-order group, while diminishing or obscuring the effect in the MI-MC first-order group, thereby working against the detection of ALS in that order group.

## Experiment 4

So far, we have analyzed the ALS in a within-participants design using the color-word Stroop task. Across three experiments, we found converging evidence that the ALS effect critically depended on the order of transitions. Moreover, both the selective presence of the ALS effect in the MC-MI first-order group and its selective absence in the MI-MC first-order group may be attributable to a combination of different types of practice. In all three experiments, a substantial RT decrease and a concurrent reduction in congruency effects were found across the experiment. Additionally, the ALS effect was only present in frequency-biased item sets, but not when stimulus-specific practice was controlled for, as in unbiased item sets.

Before jumping to conclusions, we decided to give it a final try and used more complex stimuli that might be less affected by stimulus-specific practice effects. To this end, in Experiment 4, we investigated the ALS effect with a face-name Stroop task, in which participants responded to the learned identity of six face-name combinations. Thus, this face-name Stroop task relies on arbitrary pairings of relevant (face) and irrelevant (name) information. These pairings are not processed automatically; instead, they necessitate initial learning and must be actively maintained in working memory. This increased cognitive demand may reduce the resources available for encoding and sustaining stimulus-specific contingencies (see also Wühr et al., 2015). As in Experiment 3, two of the face-name combinations served as frequency-unbiased items. To further reduce the substantial performance improvements over time, participants were required to learn six new faces and names after the first transition.

### Method

#### Participants

Due to the counterbalancing reason (24 versions, explained later), the sample size of Experiment 4 needs to be a multiple of 24. Accordingly, we included 48 participants (38 females, and three gender-diverse; *M*_*age*_ = 22.94 years, *SD* = 4.56), all of whom were students. Participants were recruited from the University of Greifswald. They received either partial course credit or €7 for their participation. Data collection took place between May 16, 2024, and June 13, 2024. Except for one participant with Polish as their mother tongue, all the others were native German speakers. All participants had normal or corrected-to-normal vision.

#### Apparatus and stimuli

The face-name Stroop task was programmed using PsychoPy (v2023.2.3; Peirce et al., 2019, 2022). The questionnaire collecting demographic data was generated using SoSci Survey (Leiner, 2024) and was made available to users via www.soscisurvey.de. The whole data collection was conducted using the open-source platform Pavlovia.org (see also Bridges et al., 2020).

Two different sets of face-name combinations were used for the first transition (e.g., set 1: Sarah, Anton, Emily, Jakob, Tabea, David) and for the second transition (e.g., set 2: Julia, Franz, Alice, Oskar, Laura, Henry), the order of sets being counterbalanced across participants. The 12 face stimuli were selected from (Langner et al., 2010; see Appendix B). The 12 names were presented in Open Sans font (letter height 0.05) in white.

The task followed a 6-AFC paradigm, and each set of six stimuli in the first and second transition (set 1, set 2) was divided into three subsets with non-overlapping stimulus and response sets (set 1: Sarah-Anton, Emily-Jakob, and Tabea-David; set 2: Julia-Franz, Alice-Oskar, and Laura-Henry). These pairings remained constant, for example, the name Sarah never appeared on the face of Jakob (see Blais & Bunge, 2010; Bugg et al., 2008; Schmidt, 2014). This design ensured that each target face was only presented either with its respective congruent name (e.g., Sarah’s face with Sarah’s name) or with a predetermined incongruent name (e.g., Sarah’s face with Anton’s name), resulting in a total of 12 unique stimuli drawn from the full 6 × 6 stimulus matrix, for each stimulus set (set 1, set 2).

Responses were made using the ring (S, L), middle (D, K), and index (F, J) fingers of both hands, with each subset of stimuli uniquely assigned to homologous finger pairs of the left and right hands (e.g., Emily was mapped to the left middle finger and Jakob to the right middle finger). As the subsets were gender-paired, we alternated gender across the response keys (i.e., from left-to-right: m-f-m-f-m-f or f-m-f-m-f-m, see Table 2).

**Table 2:** Stimulus–response (S-R) mappings for stimulus set 1

key/gender	S/m	D/f	F/m	J/f	K/m	L/f
S-R 1	**David**	Sarah	Jakob	Emily	Anton	Tabea
S-R 2	Anton	Emily	**David**	Tabea	Jakob	Sarah
S-R 3	Jakob	Tabea	Anton	Sarah	**David**	Emily
key/gender	S/f	D/m	F/f	J/m	K/f	L/m
S-R 4	Emily	Anton	Tabea	**David**	Sarah	Jakob
S-R 5	Tabea	Jakob	Sarah	Anton	Emily	**David**
S-R 6	Sarah	**David**	Emily	Jakob	Tabea	Anton

Six chosen S-R mappings for stimulus set 1 are shown with gender alternated as m-f-m-f-m-f on the upper panel (S-R 1, 2, 3), and as f-m-f-m-f-m on the lower panel (S-R 4, 5, 6). Each target stimulus is assigned to each response key across the six S-R mappings (e.g., illustrated with David printed in bold). Similarly, we have six S-R mappings for stimulus set 2

#### Procedure

The procedure of Experiment 4 closely paralleled that of Experiment 3 but differed in two aspects. First, face-name identity and the respective response mapping were acquired in three practice blocks, consisting of 36 trials for face-identity learning (six trials per face), 36 trials for name learning (six trials per name), and 24 trials for practicing S-R mappings (four trials per person identity, with face and name shown together, all congruent). Only for the first two practice blocks the person identity and corresponding S-R mapping were shown in the lower part of the screen as a learning aid. Participants could only proceed if they reached 80% accuracy in each practice block. Otherwise, the practice block was repeated. Second, as mentioned earlier, after the first transition, a new set of stimuli (i.e., six new faces and names) was used. Hence, three practice blocks with new stimuli were conducted before the second transition.

As in Experiment 3, following the practice blocks, participants completed both transitions from a PC1 block (MC or MI) to a PC2 block (MI or MC), which were separated by a 2-min distractor task. The face-name combinations from subsets 1 and 2 served as the biased items (PC-80/PC-20), and subset 3 stimuli served as the unbiased items (PC-50), which were presented equally with their congruent and predetermined incongruent names. The order of stimulus presentation within each block was pseudorandomized to prevent repetition of subsets in consecutive trials, which ensured that exact stimuli and homologous fingers were not repeated (e.g., left index finger was never followed by right index finger). The specific proportions of congruent and incongruent trials in the MC and MI blocks of each set of face-name combinations corresponded to the percentages of color-word combinations in Experiment 3 (see Table 1).

The trial procedure differs slightly from those of the previous three experiments. Each trial began with a 1,000 ms fixation interval. To induce a large enough conflict, the name (i.e., distractor) appeared 200 ms before the onset of the face (i.e., target). Then, the whole Stroop stimulus, consisting of a face and a name, remained on the screen until a response was made. Feedback was provided only for errors, with the German word “Fehler” displayed for 300 ms. After an inter-trial interval of 500 ms, during which the screen went blank, the next trial began. The entire experiment took approximately 70 min, during which RTs and ERRs were recorded.

The PC ratio, distribution of unbiased versus biased items, number of trials, etc., were the same as in Experiment 3. The experiment consisted of 24 versions after counterbalancing order of transition (MC-MI first vs. MC-MI first), order of stimulus set (set 1 vs. set 2), and S-R mappings (six ways of mapping).

#### Design

The design was identical to that of Experiment 1, except that we applied the two-step procedure separately for each item type (biased, unbiased), following the approach used in previous studies (e.g., Bugg, 2014; Bugg et al., 2011).

### Results

#### Data preprocessing

The same outlier procedure used in Experiment 1 was applied in Experiment 4. All analyses were restricted to test blocks, resulting in a dataset of 45,312 experimental trials (48 participants × 944 trials). The first trial of each block was excluded (0.42% of trials). Based on the outlier criteria, data from 44 participants were included in the final analyses (see Tukey, 1977). The RT analysis was restricted to correct responses, excluding errors (4.71% of trials) and post-error trials (4.24% of trials). Additionally, extreme RTs were excluded (0.11% of trials; see J. Miller, 2023).

#### Biased items (PC-80 and PC-20 items)

##### RT data

The 2 × 2 × 2 × 2 mixed-factors ANOVA on RTs of biased items with Order of Transition as a between-participants factor and Transition, Block, and Congruency as within-participants factors revealed a significant congruency effect, *F*(1, 42) = 145.60, *p* <.001, ɳ_p_^2^ =.78, with longer RTs for incongruent trials (*M* = 797 ms ± *SD* = 124) compared to congruent trials (*M* = 704 ms ± *SD* = 116). The congruency effect was further modulated by a significant interaction with Transition and Block, *F*(1, 42) = 78.79, *p* <.001, ɳ_p_^2^ =.65, showing an overall LWPC effect. The post hoc analysis revealed a substantial decrease in the congruency effect for the MC-MI transition, *F*(1, 42) = 66.42, *p* <.001, ɳ_p_^2^ =.61 (mean congruency effects of 170 ms and 50 ms for PC1 and PC2 blocks, respectively) compared to an increase in the congruency effect for the MI-MC transition, *F*(1, 42) = 44.33, *p* <.001, ɳ_p_^2^ =.51 (mean congruency effects of 42 ms and 110 ms for PC1 and PC2 blocks, respectively). Notably, and in contrast to Experiments 1–3, neither the interaction between Order of Transition and Transition, *F*(1, 42) = 1.17, *p* =.285, ɳ_p_^2^ =.03 – which would have indicated generally faster responses in the second transition compared to the first – nor the interaction between Order of Transition, Transition, and Congruency, *F* < 1 – which would have indicated a reduction in congruency effects from the first to the second transition – were significant. Finally, a significant four-way interaction was observed, *F*(1, 42) = 6.25, *p* =.016, ɳ_p_^2^ =.13, suggesting that the pattern of decreasing congruency effects in MC-MI transitions and increasing congruency effects in MI-MC transitions depended on the order of transitions (i.e., more pronounced in the MC-MI first-order group).

In contrast to Experiment 3, the subsequent 2 × 2 × 2 mixed-factors ANOVA on congruency effects using Order of Transition as a between-participants factor and Transition and PC as within-participants factors revealed an ALS effect, indicated by the significant interaction between Transition and PC, *F*(1, 42) = 13.58, *p* <.001, ɳ_p_^2^ =.24: The decrease in the congruency effect for the MC-MI transition (120 ms; MC minus MI) was larger than the increase for the MI-MC transition (68 ms; MC minus MI; see Fig. 2, Sect. 4, left panel). The interaction between Transition and PC was not further modulated by the Order of Transition, *F* < 1, suggesting an ALS effect for both order groups. Still, given that the ALS had been dependent on the Order of Transition in all previous experiments, we looked into each order group separately, and, indeed, the ALS was significant for both transition orders: For the MC-MI first-order group, the interaction between Transition and PC was significant, *F*(1, 22) = 6.58, *p* =.018, ɳ_p_^2^ =.23, revealing an ALS effect: The decrease in the congruency effect in the MC-MI transition (146 ms; MC minus MI) was larger than the increase of congruency effect in the MI-MC transition (92 ms; MC minus MI). In accordance with this, for the MI-MC first-order group, the interaction between Transition and PC was also significant, *F*(1, 20) = 7.65, *p* =.012, ɳ_p_^2^ =.28, which again indicates an ALS effect: The decrease in the congruency effect in the MC-MI transition (90 ms; MC minus MI) larger than the increase of congruency effect in the MI-MC transition (43 ms; MC minus MI) (see Fig. 3, Sect. 4.1A).

##### Error data

The overall ERR for biased items was 5.56%. The corresponding 2 × 2 × 2 × 2 mixed-factors ANOVA on ERRs of biased items revealed a significant congruency effect, *F*(1, 42) = 42.93, *p* <.001, ɳ_p_^2^ =.51, with more errors in incongruent trials (*M* = 6.97% ± *SD* = 6.32) than congruent ones (*M* = 4.14% ± *SD* = 4.82). In addition, this congruency effect was modulated by a significant interaction with Transition and Block, *F*(1, 42) = 30.21, *p* <.001, ɳ_p_^2^ =.42. Post hoc analyses revealed a substantial decrease in the congruency effect for the MC-MI transition, *F*(1, 42) = 37.92, *p* <.001, ɳ_p_^2^ =.47 (mean congruency effects of 5.75% and 0.39% for the PC1 and PC2 blocks, respectively), compared to an increase in the congruency effect for the MI-MC transition, *F*(1, 42) = 5.87, *p* =.020, ɳ_p_^2^ =.12 (mean congruency effects of 1.49% and 3.69% for PC1 and PC2 blocks, respectively).

As in the RT analysis, the subsequent 2 × 2 × 2 mixed-factors ANOVA on congruency effects revealed an ALS effect, indicated by the significant interaction between Transition and PC, *F*(1, 42) = 7.49, *p* =.009, ɳ_p_^2^ =.15: The decrease in the congruency effect for the MC-MI transition (5.36%; MC minus MI) was larger than the increase for the MI-MC transition (2.20%; MC minus MI). The three-way interaction between the Order of Transition, Transition, and PC was not statistically significant, *F* < 1. For the MC-MI first-order group, the interaction between Transition and PC was significant, *F*(1, 22) = 6.99, *p* =.015, ɳ_p_^2^ =.24, revealing an ALS effect: The decrease in the congruency effect in the MC-MI transition (6.13%; MC minus MI) was larger than the increase of congruency effect in the MI-MC transition (1.93%; MC minus MI). For the MI-MC first-order group, the interaction between Transition and PC was not significant, *F*(1, 20) = 1.54, *p* =.229, ɳ_p_^2^ =.07.

#### Unbiased items (PC-50 items)

##### RT data

The analogous 2 × 2 × 2 × 2 mixed-factors ANOVA on RTs of unbiased items with Order of Transition as a between-participants factor and Transition, Block, and Congruency as within-participants factors showed a significant congruency effect, *F*(1, 42) = 181.74, *p* <.001, ɳ_p_^2^ =.81, with longer RTs for incongruent trials (*M* = 797 ms ± *SD* = 119) than congruent trials (*M* = 698 ms ± *SD* = 114). This congruency effect was further modulated by a significant interaction with Transition and Block, *F*(1, 42) = 15.60, *p* <.001, ɳ_p_^2^ =.27, showing an overall LWPC effect also for unbiased items. The post hoc analysis revealed a significant decrease in the congruency effect for the MC-MI transition, *F*(1, 42) = 18.45, *p* <.001, ɳ_p_^2^ =.30 (mean congruency effects of 153 ms and 65 ms for PC1 and PC2 blocks, respectively) compared to an insignificant increase in the congruency effect for the MI-MC transition, *F*(1, 42) = 1.56, *p* =.219, ɳ_p_^2^ =.04 (mean congruency effects of 79 ms and 100 ms for PC1 and PC2 blocks, respectively). The interaction between Order of Transition and Transition was significant, indicating that for unbiased items, participants responded slightly faster in the second than in the first transition, *F*(1, 42) = 4.66, *p* =.037, ɳ_p_^2^ =.10. The interaction between Order of Transition, Transition, and Congruency was not significant, *F* < 1, suggesting that congruency effects did not overall decline from the first to the second transition. The significant four-way interaction, *F*(1, 42) = 10.17, *p* =.003, ɳ_p_^2^ =.20, mirrored the finding for biased items, suggesting that the pattern of decreasing congruency effects in MC-MI transitions and increasing congruency effects in MI-MC transitions depended on the order of transitions (i.e., more pronounced in the MC-MI first-order group).

The subsequent 2 × 2 × 2 mixed-factors ANOVA on congruency effects using the Order of Transition as a between-participants factor and Transition and PC as within-participants factors revealed a significant interaction between Transition and PC, *F*(1, 42) = 6.98, *p* =.012, ɳ_p_^2^ =.14, indicating an ALS effect: The decrease of the congruency effect in the MC-MI transition (88 ms; MC minus MI) was larger than the increase of congruency effect in the MI-MC transition (21 ms; MC minus MI; see Fig. 2, Sect. 4, right panel). The three-way interaction between the Order of Transition, Transition, and PC was not statistically significant, *F*(1, 42) = 1.14, *p* =.293, ɳ_p_^2^ =.03. For the MC-MI first-order group, we again observed an ALS effect, i.e., the interaction between Transition and PC was significant, *F*(1, 22) = 7.89, *p* =.010, ɳ_p_^2^ =.26: The decrease in the congruency effect in the MC-MI transition (141 ms; MC minus MI) was larger than the increase in the MI-MC transition (50 ms; MC minus MI). For the MI-MC first-order group, the interaction between Transition and PC failed to reach significance, *F*(1, 20) = 1.08, *p* =.310, ɳ_p_^2^ =.05, indicating no ALS effect (see Fig. 3, Sect. 4.2A).

##### Error data

The overall ERR for unbiased items was 6.14%. The corresponding 2 × 2 × 2 × 2 mixed-factors ANOVA on ERRs of unbiased items revealed a significant congruency effect, *F*(1, 42) = 21.08, *p* <.001, ɳ_p_^2^ =.33, with more errors in incongruent trials (*M* = 7.58% ± *SD* = 8.45) compared to congruent trials (*M* = 4.71% ± *SD* = 7.28), which was not further modulated by any other factor, all *F*s ≤ 3.61, all *p*s ≥.064, all ɳ_p_^2^s ≤.08.

The subsequent 2 × 2 × 2 mixed-factors ANOVA on congruency effects also revealed no evidence for an ALS effect, *F* < 1, which was not affected by the Order of Transition, *F* < 1. No ALS effects were observed for either the MC-MI first-order group, *F* < 1, or the MI-MC first-order group, *F* < 1.

### Discussion

Using a face-name Stroop paradigm, Experiment 4 successfully revealed an ALS effect on RTs for both biased and unbiased items. We specifically aimed to further reduce stimulus-specific learning by using more complex stimuli (human faces and names) and to reduce the general RT decrease and the concurrent reduction in congruency effects over time by introducing new faces and names prior to the second transition. An ALS effect, particularly for frequency-unbiased items, suggests that the asymmetry of the stronger congruency decrease in the MC-MI transition than the increase in the MI-MC transition is related to a control adjustment rather than reflecting an artifact of mere practice.

However, considering the order of transitions made the result pattern more complex. The ALS effect was found in biased items, irrespective of the order of transition. Although there was statistically no difference in the ALS effect between the two order groups in unbiased items, it remained statistically significant for the MC-MI first-order group but was absent in the MI-MC first-order group.

## General discussion

Across four experiments, we investigated whether the ALS effect – originally reported by Abrahamse et al. (2013) in a between-participants design – also appears using a within-participants design. This approach enabled us to investigate whether prior experience with one type of transition (e.g., MC-MI) affects adaptation during the opposite transition (e.g., MI-MC). To this end, each participant worked through both transitions from a PC1 block (MC or MI) to a PC2 block (MI or MC). Our results show that the ALS effect can be observed in a within-participants design. Experiments 1 and 4 provide strong overall evidence, while Experiments 2 and 3 offer partial support, particularly for the MC-MI first-order group: The decrease in the congruency effect during the MC-MI transition was significantly larger than the increase during the MI-MC transition.

Across the biased items in all three color-word Stroop task experiments, it became apparent that the overall pattern of the ALS effect depended on the order of transitions and was almost entirely driven by the group that experienced the MC-MI transition first (i.e., MC-MI first-order group; see Fig. 3, Sect. 1A-3.1A, left panel). In contrast, the ALS effect was virtually absent in the group that started with the reversed transition (i.e., MI-MC first-order group; see Fig. 3, Sect. 1A-3.1A, right panel). As a possible explanation for the consistent asymmetry in ALS, we identified different types of practice effects (see Schmidt, 2016) that, in combination with the additional task exposure inherent in the within-participants design, either support or oppose the ALS, depending on the order of transitions.

### The influence of practice effects

The increased task exposure revealed a strong and consistent pattern across all three color-word Stroop task experiments, highlighting the significant and reliable influence of nonspecific practice benefits over time and the concurrent advantage for the second transition: Participants responded consistently faster (as reflected by the significant interactions between Order of Transition and Transition), and congruency effects were consistently smaller (as indicated by the significant interactions between Order of Transition, Transition, and Congruency) in the second transition relative to the first. Consequently, the combination of these different practice benefits may either support or oppose the ALS effect (see Schmidt, 2016), depending on the order of transitions.

In the MC-MI first-order group, frequent trial types and stimulus-specific learning produced a pronounced reduction in RTs for the frequent incongruent trials in MI blocks during the MC-MI transition (see Fig. 3, Section B, left panel). These effects were accompanied by general practice benefits. Thus, both forms of practice converged to decrease the congruency effect during the first transition (MC-MI). However, during the second transition (MI-MC), these influences worked in opposite directions: Whereas the occurrence of frequent congruent trials and rare incongruent trials in MC blocks should increase congruency effects, general practice benefits should instead reduce them (see Schmidt, 2016). Importantly, this leads to an exaggeration of the ALS pattern in the MC-MI first-order group.

However, in the MI-MC first-order group, these same practice-related benefits may even hinder the development of an ALS pattern altogether. The substantial general RT decrease by the time participants reach the second transition (MC-MI) prevents a similarly strong decrease in the congruency effect when shifting from MC to MI blocks, as seen when the same transition is performed at the start of the experiment (i.e., in the MC-MI first-order group). Similarly, if the MI-MC transition is performed first, the increase in congruency effects from the MI to the MC block may be greater, as it is less affected by general practice benefits than when performed as the second transition (see Fig. 3, Section B, right panel). Accordingly, in the MI-MC first-order group, these same practice-related benefits produce a smaller decrease of congruency effects during the MC-MI transition and a larger increase during the MI-MC transition, compared with the MC-MI first-order group. As a result, they operate in the opposite direction of an ALS pattern, thereby diminishing or obscuring the effect in the MI-MC first-order group and potentially working against the detection of ALS in that direction.

### Minimizing practice-related confounding factors

Throughout the three experiments involving the color-word Stroop task, we identified various forms of practice – including trial-type and stimulus-specific practice, general RT decrease, and the decrease in congruency effects over time – that can either facilitate or obscure the ALS effect (see Schmidt, 2016), depending on the order in which participants experienced the transitions. These competing influences likely account for the consistent asymmetries observed across the experiments. In Experiment 4, we successfully minimized these practice-related confounding factors. To minimize stimulus-specific learning, we employed more complex stimuli – human faces paired with names. Unlike the color-word Stroop task used in Experiments 1–3, which involves strongly overlearned associations between the relevant (color) and irrelevant (word) dimensions of the task, the face-name Stroop task in Experiment 4 relies on arbitrary pairings of relevant (face) and irrelevant (name) information. These pairings are not processed automatically; instead, they require deliberate learning and must be kept active in working memory. This increased cognitive demand can limit the resources available for encoding and maintaining stimulus-specific contingencies (see also Wühr et al., 2015). Additionally, to address the general decrease in RT and the observed decrease in congruency effects over time, we introduced a new set of six faces and names prior to the second transition. This method ensured a similar potential for RT changes across both transitions, regardless of the order.

For biased items, these methodological modifications revealed a strong ALS effect, regardless of the order of transitions. Nonspecific practice effects were effectively removed, as evidenced by the near-complete absence of general RT reductions across the task (as shown by the non-significant interaction between Order of Transition and Transition) and the lack of a significant decrease in congruency effects with practice (no significant interaction between Order of Transition, Transition, and Congruency). Still, a strong ALS effect was observed in RTs. Importantly, the ALS effect was evident not only in the MC-MI first-order group, as demonstrated in previous experiments, but also, for the first time, in the MI-MC first-order group. Crucially, the ALS effect observed in RTs for frequency-unbiased items suggests that the asymmetry, characterized by a stronger decrease in congruency during the MC-MI transition compared to the increase during the MI-MC transition, is linked to a control adjustment rather than being an artifact of practice. Overall, the findings of Experiment 4 suggest that when practice effects are properly controlled for, an ALS effect can be reliably observed irrespective of order conditions, especially for biased items.

### Implications for the (in-)flexibility of control adaptations

According to Abrahamse et al. (2013), shifting from an MC block to an MI block does not produce the same effects as shifting in the opposite direction, with a larger decrease in the congruency effect during the MC-MI transition than the increase during the MI-MC transition. This ALS effect can be explained by the attention modulation account, which predicts this pattern in response to the LWPC manipulation (e.g., Abrahamse et al., 2013; Lindsay & Jacoby, 1994; Logan & Zbrodoff, 1979; Lowe & Mitterer, 1982). Since interference is high during an MI block, attention is focused on the task-relevant dimension. This focus not only reduces the congruency effect but also prevents participants from noticing the shift in PC when the MC block starts (Abrahamse et al., 2013). The extent of this inflexibility in control adaptations was also demonstrated by Abrahamse et al. (2013) in their Experiment 2, where participants were first trained with either MC or MI blocks and then tested on the MI-MC or MC-MI transitions, respectively. In both groups, the results showed that in the MI condition, attention was intensely focused on the task-relevant dimension, as indicated by the lack of significant changes in the congruency effect after exposure to an MI block: The congruency effect did not significantly vary across all three phases in the MI-MC-MI group, and it did not significantly increase from MI to MC in the MC-MI-MC group. These findings support the idea that exposure to high-conflict contexts can lead to a rigid control state, limiting future adaptation and highlighting the inflexibility of control adaptations once a shielding strategy is adopted.

Importantly, in Experiment 4, where we minimized practice-related confounding factors by using more complex stimuli and a completely new stimulus set in the second transition, we observed a significant ALS effect for both the MC-MI first-order group and the MI-MC first-order group. In both cases, the decrease in the congruency effect during the MC-MI transition consistently exceeded the increase observed during the MI-MC transition, regardless of the order in which these transitions occurred. Crucially, our study presents a novel finding: While different shift directions lead to asymmetric effects (i.e., the re-adaptation is stronger in MC-MI than in MI-MC transitions), prior experience with one type of transition (e.g., MC-MI) does not seem to affect adaptation during the opposite transition (e.g., MI-MC) when confounding practice effects are minimized. Unlike the results reported by Abrahamse et al. (2013) for their MI-MC-MI group – where the congruency effect remained stable across blocks – we observed a significant change in congruency effects in the comparable MI-MC first-order group (i.e., MI-MC transition followed by MC-MI transition) in our within-participants design in Experiment 4. This indicates that participants were indeed able to detect the change in PC in the subsequent MC block in the second transition and adjust their control strategies accordingly, given that the context shift was made more obvious through the introduction of new stimulus material. Under these conditions, participants’ control state seems to be “reset,” allowing them to break free from the previously established attentional focus on the relevant dimension. As a result, they became more responsive to the altered context, and the ability to adapt to the new task demands – and benefit from the change in PC – was restored at the beginning of the second transition. In other words, participants could start fresh and flexibly reconfigure their control settings in response to different environments that require distinct optimal strategies instead of the continuing usage of outdated yet once-effective processing strategies. In the deer-crossing analogy presented earlier, this indicates that participants were able to break free from the shielding control state and fully adapt their behavior in response to the fence when driving on a different road. This finding builds on previous research, showing that the presumed inflexibility of control adaptation is actually more flexible than previously thought. Specifically, participants do not necessarily “lock in” their control settings after encountering a MI block, nor do they become insensitive to subsequent context shifts, as long as certain contextual conditions (e.g., minimizing practice effects, salient context change by the introduction of new stimuli) are met.

Although empirical evidence for the ALS effect remains limited, one recent study reported robust evidence for asymmetrical list shifting. Specifically, Kelber et al. (2025) mostly observed significant ALS effects for both frequency-biased and -unbiased items across three experiments using different versions of a manual counting Stroop task. Importantly, however, the ALS effects on RTs were statistically significant in only two of the three experiments. In these two experiments, the authors found no practice effects (as evidenced by the non-significant interactions between congruency and practice) and therefore argued that the observed ALS effects could not be fully accounted for by item-specific learning or contingency learning mechanisms. Together with our findings, this suggests that it may depend on the specific tasks and stimuli used, whether participants rely on associative learning or use proactive control when faced with PC contexts, a strategy termed “associations-as-antagonists-to control” account (Bugg, 2014), or shortly “last-resort” account (see Bugg, 2014; Spinelli & Lupker, 2023). In other words, participants may rely on proactive control when item-specific or contingency learning appears less efficient. Although Kelber et al. (2025) had already demonstrated an ALS effect while explicitly testing for practice in a between-participants design, the present study extends these findings by providing more systematic evidence. Specifically, by employing a within-participants design, we were able to examine the effect with greater sensitivity and to more precisely delineate which types of practice effects may contribute to or confound the observed ALS effect. Our results are less consistent with the framework proposed by Spinelli and Lupker (2023), who suggested that the simultaneous maintenance of multiple arbitrary S-R-associations may place such high demands on cognitive resources that proactive control adaptation processes cannot be effectively engaged. At least the results of our Experiment 4 are hard to reconcile with this view, given that human faces and names had to be learnt from scratch and participants had to learn completely new S-R associations during the second transition. If anything, this suggests that the increased working memory load reduced item-specific and contingency learning, pushing participants towards a proactive control strategy (resulting in an ALS effect for both biased and unbiased items).

### Limitations and suggestions for future directions

Our study of the ALS effect using a within-participants design, however, also has certain limitations. Notably, we cannot definitively rule out the possibility that factors other than conflict adaptation, at least in part, contributed to the observed effects. As argued by Schmidt (2016), benefits of practice, such as a reduction in congruency effects, can already appear from Block 1 to Block 2 within a single transition. Since residual effects indicate that practice alone may not fully explain the ALS findings by Abrahamse et al. (2013), leaving room for mechanisms like conflict adaptation (Schmidt, 2016), we focused on reducing practice-related confounds between transitions by introducing a new stimulus set between transitions. While this manipulation helps reduce lower-level repetition and contingency learning *between* transitions (i.e., from MC-MI to MI-MC and vice versa), it does not eliminate them entirely *within* transitions (i.e., from MC to MI and vice versa). Future research may want to examine whether using completely new stimulus sets in each block would further affect the observed result patterns.

In the present study, we have demonstrated the ALS effect using a full within-participants design. Across experiments, we identified multiple forms of practice – including trial-type and stimulus-specific learning, general reductions in RT, and a decrease in congruency effects over time – that have the potential to either enhance or obscure the ALS effect, depending on the order of transitions. Importantly, when practice-related confounds were minimized between transitions, the ALS effect appeared, regardless of the order of transition. Given that the ALS effect is a marker of inflexible control adaptation, our findings suggest that a salient context change may overcome this inflexibility. In Experiment 4, we succeeded in reducing the practice effects that obscured the ALS *between* transitions (from MC-MI to MI-MC and vice versa). This leaves room for the speculation that the same context change, like using a new stimulus set *within* a transition (from MI to MC and vice versa), may potentially even prevent the ALS effect and restore flexible context adaptation.

## Supplementary Information

Below is the link to the electronic supplementary material.

Supplementary file1 (DOCX 320 KB)

## Data Availability

Trial-level data for all experiments in this article are available at the following link: 10.5283/epub.79353. Additional study materials (PsychoPy experiment files and R analysis scripts) can be obtained by contacting the corresponding author by email.

## Footnotes

1 Alternative accounts have been proposed to explain the LWPC effect on the basis of item-specific control (e.g., Blais & Bunge, 2010; Bugg et al., 2008) or item-specific contingency learning, which is driven by the predictive relationships between words and responses (Schmidt et al., 2007; Schmidt, 2013; Schmidt & Besner, 2008; for a review, see Schmidt, 2019). To address these influences, researchers have embedded additional sets of frequency-unbiased stimuli to isolate the effects of cognitive control (e.g., Bugg et al., 2011). Although there is still an ongoing debate about which account best explains the LWPC effect (for a review, see Bugg, 2012), it is conceivable that multiple influences are involved (see Surrey et al., 2019).

2 Note that Abrahamse et al. (2013) had already observed within-participant asymmetric list shifting in Experiment 2 using an ISPC manipulation (Jacoby et al., 2003) in which attentional modulation is driven by the proportion congruency of specific items rather than the entire block. However, their design did not include full within-participant transitions for both MC-MI and MI-MC, as participants were initially trained on either MC or MI items and then tested on the MI-MC or MC-MI transitions, respectively.

3 Note that due to a programming error in Experiments 1 and 2, the distribution of congruent and incongruent trials deviates slightly from the intended 80% to 20% congruency ratio in MC and MI blocks, respectively.

4 Note that the interaction between Transition, Block, and Congruency simultaneously reflects whether a significant LWPC effect was present. Because the three-way interaction shows a decrease of congruency effects from MC to MI blocks and an increase from MI to MC blocks, congruency effects are generally smaller in MI than in MC blocks, thus reflecting an LWPC effect (see Fig. 2, Sect. 1).

5 In the LWPC paradigm of Experiments 1 and 2, trial-type practice cannot be distinguished from stimulus-specific practice. Trial-type practice refers to the differing frequency of congruent and incongruent trials in MC and MI blocks. Stimulus-specific practice refers to the frequency of specific stimuli (e.g., percentage of times the word *RED* is congruent in MC and MI blocks). For example, the frequency of the word *RED* in red color is confounded with the frequency of congruent trials in the MC block. We address this issue in Experiments 3 and 4 when using frequency-unbiased items (for a review, see Bugg, 2012).

6 The general decrease of congruency effects between sessions (i.e., between the first and the second transition) is reflected in the significant interaction between Order of Transition, Transition, and Congruency, *F*(1, 47) = 21.13, *p* <.001, ɳ_p_^2^ =.31 (see Table A1 in the Appendix). Applying the same logic as for the interaction between Order of Transition and Transition, any differences in congruency effects between transition types – regardless of when they occurred – were modulated by a significant interaction with the order of transition. This demonstrates that it is the temporal position of the transition, rather than solely its type, that drives the difference in congruency effects.

## Code availability

All analyses were performed using R Statistical Software (v4.3.1; R Core Team, 2021) and the packages *tidyverse* (v2.0.0; Wickham et al., 2019) and *rstatix* (v0.7.2; Kassambara, 2023). The analysis scripts can be obtained by contacting the corresponding author by email.

## Declarations

### Open practices statement

Trial-level data for all experiments in this article are available at the following link: 10.5283/epub.79353. Additional study materials (PsychoPy experiment files and R analysis scripts) can be obtained by contacting the corresponding author by email.

Experiment 1 was initially conducted as a pilot study and was therefore not preregistered. In contrast, Experiment 2 (https://aspredicted.org/jwvt-mpdc.pdf), Experiment 3 (https://aspredicted.org/jmrt-kmt7.pdf), and Experiment 4 (https://aspredicted.org/ctzj-t3fn.pdf) were preregistered. Please note that the second analysis in the two-step procedure was not included in the original preregistration. We deviated from the initial plan by incorporating this second-step analysis to specifically test for the asymmetry of the adaptation, namely, that the decrease in the congruency effect during the MC-MI transition would be significantly larger than the increase observed during the MI-MC transition. Except for this second-step analysis, all other analyses followed the preregistered protocol.
