# Supplementary material for: The asymmetric list shift effect – flexible adaptation to new context demands?
Source: Atten Percept Psychophys. 2026 Jun 9;88(5):142. doi: 10.3758/s13414-026-03284-x (PMC13249780; doi:10.3758/s13414-026-03284-x)

# Appendix A

# Results of the mixed-measures ANOVAs

Table A1 All effects of the 2 × 2 × 2 × 2 mixed-factors ANOVA on response times (RTs) and error rates (ERRs) with Order of Transition as a between-participants factor and Transition, Block, and Congruency as within-participants factors

| Exp. | RT effect | *F* | *p* | ɳ_p_^2^ |  | ERR effect | *F* | *p* | ɳ_p_^2^ |
| --- | --- | --- | --- | --- | --- | --- | --- | --- | --- |
| 1 | O | 0.01 | .907 | .00 |  | O | 0.14 | .707 | .00 |
|  | T | 0.51 | .477 | .01 |  | T | 1.91 | .173 | .04 |
|  | B | 15.49 | < .001 | .25 |  | B | 3.52 | .067 | .07 |
|  | C | 132.93 | < .001 | .74 |  | C | 13.09 | < .001 | .22 |
|  | O × T | 129.87 | < .001 | .73 |  | O × T | 2.62 | .112 | .05 |
|  | O × B | 1.80 | .186 | .04 |  | O × B | 4.76 | .034 | .09 |
|  | O × C | 0.01 | .909 | .00 |  | O × C | 1.32 | .257 | .03 |
|  | T × B | 4.93 | .031 | .10 |  | T × B | 13.16 | < .001 | .22 |
|  | T × C | 4.60 | .037 | .09 |  | T × C | 0.09 | .764 | .00 |
|  | B × C | 6.03 | .018 | .11 |  | B × C | 1.81 | .185 | .04 |
|  | O × T × B | 5.04 | .029 | .10 |  | O × T × B | 0.32 | .575 | .01 |
|  | O × T × C | 21.13 | < .001 | .31 |  | O × T × C | 0.22 | .640 | .01 |
|  | O × B × C | 16.61 | < .001 | .26 |  | O × B × C | 0.86 | .358 | .02 |
|  | T × B × C | 50.86 | < .001 | .52 |  | T × B × C | 25.75 | < .001 | .35 |
|  | O × T × B × C | 1.73 | .195 | .04 |  | O × T × B × C | 1.65 | .206 | .03 |
| 2 | O | 0.00 | .984 | .00 |  | O | 0.03 | .874 | .00 |
|  | T | 1.35 | .252 | .03 |  | T | 2.07 | .157 | .04 |
|  | B | 3.92 | .053 | .08 |  | B | 0.12 | .729 | .00 |
|  | C | 72.66 | < .001 | .61 |  | C | 5.84 | .020 | .11 |
|  | O × T | 24.02 | < .001 | .34 |  | O × T | 0.00 | .989 | .00 |
|  | O × B | 1.89 | .176 | .04 |  | O × B | 0.39 | .537 | .01 |
|  | O × C | 0.01 | .927 | .00 |  | O × C | 1.80 | .186 | .04 |
|  | T × B | 4.39 | .042 | .09 |  | T × B | 2.29 | .136 | .05 |
|  | T × C | 4.46 | .040 | .09 |  | T × C | 1.99 | .165 | .04 |
|  | B × C | 3.45 | .070 | .07 |  | B × C | 0.10 | .760 | .00 |
|  | O × T × B | 2.18 | .147 | .04 |  | O × T × B | 3.04 | .088 | .06 |
|  | O × T × C | 13.17 | < .001 | .22 |  | O × T × C | 0.55 | .460 | .01 |
|  | O × B × C | 3.85 | .056 | .08 |  | O × B × C | 0.25 | .617 | .01 |
|  | T × B × C | 21.93 | < .001 | .32 |  | T × B × C | 39.82 | < .001 | .46 |
|  | O × T × B × C | 0.32 | .573 | .01 |  | O × T × B × C | 0.24 | .629 | .01 |
| 3.1 | O | 2.45 | .124 | .05 |  | O | 0.09 | .765 | .00 |
|  | T | 0.30 | .585 | .01 |  | T | 0.06 | .809 | .00 |
|  | B | 26.20 | < .001 | .36 |  | B | 0.28 | .599 | .01 |
|  | C | 133.25 | < .001 | .74 |  | C | 11.31 | .002 | .19 |
|  | O × T | 63.29 | < .001 | .57 |  | O × T | 0.03 | .872 | .00 |
|  | O × B | 5.20 | .027 | .10 |  | O × B | 0.97 | .330 | .02 |
|  | O × C | 0.17 | .681 | .00 |  | O × C | 0.86 | .358 | .02 |
|  | T × B | 1.92 | .173 | .04 |  | T × B | 1.40 | .243 | .03 |
|  | T × C | 1.11 | .297 | .02 |  | T × C | 3.21 | .080 | .06 |
|  | B × C | 3.19 | .080 | .06 |  | B × C | 2.53 | .118 | .05 |
|  | O × T × B | 11.50 | .001 | .20 |  | O × T × B | 1.42 | .240 | .03 |
|  | O × T × C | 10.48 | .002 | .18 |  | 0 × T × C | 7.08 | .011 | .13 |
|  | O × B × C | 19.61 | < .001 | .29 |  | O × B × C | 2.04 | .160 | .04 |
|  | T × B × C | 51.30 | < .001 | .52 |  | T × B × C | 1.86 | .180 | .04 |
|  | O × T × B × C | 0.43 | .515 | .01 |  | O × T × B × C | 3.20 | .080 | .06 |
| 3.2 | O | 0.39 | .536 | .01 |  | O | 0.39 | .534 | .01 |
|  | T | 0.25 | .622 | .01 |  | T | 0.14 | .706 | .00 |
|  | B | 5.44 | .024 | .10 |  | B | 3.51 | .067 | .07 |
|  | C | 31.96 | < .001 | .41 |  | C | 1.24 | .272 | .03 |
|  | O × T | 16.81 | < .001 | .26 |  | O × T | 0.46 | .502 | .01 |
|  | O × B | 1.59 | .213 | .03 |  | O × B | 0.00 | .994 | .00 |
|  | O × C | 4.06 | .050 | .08 |  | O × C | 0.00 | .952 | .00 |
|  | T × B | 0.01 | .924 | .00 |  | T × B | 0.71 | .403 | .01 |
|  | T × C | 1.22 | .276 | .03 |  | T × C | 1.66 | .204 | .03 |
|  | B × C | 0.31 | .578 | .01 |  | B × C | 0.08 | .781 | .00 |
|  | O × T × B | 4.22 | .046 | .08 |  | O × T × B | 0.62 | .433 | .01 |
|  | O × T × C | 7.78 | .008 | .14 |  | 0 × T × C | 2.73 | .105 | .06 |
|  | O × B × C | 0.02 | .883 | .00 |  | O × B × C | 0.00 | .968 | .00 |
|  | T × B × C | 0.28 | .596 | .01 |  | T × B × C | 1.26 | .267 | .03 |
|  | O × T × B × C | 0.10 | .755 | .00 |  | O × T × B × C | 0.02 | .899 | .00 |
| 4.1 | O | 0.05 | .827 | .00 |  | O | 0.56 | .458 | .01 |
|  | T | 2.46 | .124 | .06 |  | T | 4.69 | .036 | .10 |
|  | B | 1.07 | .306 | .03 |  | B | 3.45 | .070 | .08 |
|  | C | 145.60 | < .001 | .78 |  | C | 42.93 | < .001 | .51 |
|  | O × T | 1.17 | .285 | .03 |  | O × T | 1.78 | .190 | .04 |
|  | O × B | 2.52 | .120 | .06 |  | O × B | 0.39 | .533 | .01 |
|  | O × C | 0.23 | .637 | .01 |  | O × C | 0.27 | .607 | .01 |
|  | T × B | 1.23 | .274 | .03 |  | T × B | 1.82 | .184 | .04 |
|  | T × C | 16.82 | < .001 | .29 |  | T × C | 0.51 | .479 | .01 |
|  | B × C | 13.58 | < .001 | .24 |  | B × C | 7.49 | .009 | .15 |
|  | O × T × B | 21.24 | < .001 | .34 |  | O × T × B | 0.46 | .500 | .01 |
|  | O × T × C | 0.41 | .523 | .01 |  | O × T × C | 4.39 | .042 | .10 |
|  | O × B × C | 0.07 | .789 | .00 |  | O × B × C | 0.95 | .335 | .02 |
|  | T × B × C | 78.79 | < .001 | .65 |  | T × B × C | 30.21 | < .001 | .42 |
|  | O × T × B × C | 6.25 | .016 | .13 |  | O × T × B × C | 0.15 | .704 | .00 |
| 4.2 | O | 0.25 | .622 | .01 |  | O | 0.17 | .687 | .00 |
|  | T | 1.90 | .175 | .04 |  | T | 0.70 | .407 | .02 |
|  | B | 1.07 | .307 | .03 |  | B | 2.70 | .108 | .06 |
|  | C | 181.74 | < .001 | .81 |  | C | 21.08 | < .001 | .33 |
|  | O × T | 4.66 | .037 | .10 |  | O × T | 1.68 | .202 | .04 |
|  | O × B | 3.79 | .058 | .08 |  | O × B | 1.25 | .271 | .03 |
|  | O × C | 1.69 | .200 | .04 |  | O × C | 0.45 | .507 | .01 |
|  | T × B | 0.26 | .617 | .01 |  | T × B | 0.46 | .502 | .01 |
|  | T × C | 1.68 | .202 | .04 |  | T × C | 1.31 | .259 | .03 |
|  | B × C | 6.98 | .012 | .14 |  | B × C | 0.34 | .564 | .01 |
|  | O × T × B | 11.46 | .002 | .21 |  | O × T × B | 3.61 | .064 | .08 |
|  | O × T × C | 0.16 | .690 | .00 |  | 0 × T × C | 0.44 | .510 | .01 |
|  | O × B × C | 1.14 | .293 | .03 |  | O × B × C | 0.01 | .924 | .00 |
|  | T × B × C | 15.60 | < .001 | .27 |  | T × B × C | 0.11 | .746 | .00 |
|  | O × T × B × C | 10.17 | .003 | .20 |  | O × T × B × C | 0.61 | .438 | .01 |

*Note*. The number within Exp. represents the corresponding experiment, with Experiments 3 and 4 subdivided into 3.1/4.1 for biased items and 3.2/4.2 for unbiased items; Exp. = Experiment; RT = response time; ERR = error rate; O = Order of Transition (MC-MI first, MI-MC first); T = Transition (MC-MI, MI-MC); B = Block (PC1, PC2); C = Congruency (congruent, incongruent); I = Item Type (biased, unbiased); CE = congruency effect

Table A2 All effects of the 2 × 2 × 2 mixed-factors ANOVA on congruency effects with Order of Transition as a between-participants factor and Transition and PC as within-participants factors

| Exp. | RT effect | *F* | *p* | ɳ_p_^2^ |  | ERR effect | *F* | *p* | ɳ_p_^2^ |
| --- | --- | --- | --- | --- | --- | --- | --- | --- | --- |
| 1 | O | 0.01 | .909 | .00 |  | O | 1.32 | .257 | .03 |
|  | T | 4.60 | .037 | .09 |  | T | 0.09 | .764 | .00 |
|  | PC | 50.86 | < .001 | .52 |  | PC | 25.75 | < .001 | .35 |
|  | O × T | 21.13 | < .001 | .31 |  | O × T | 0.22 | .640 | .01 |
|  | O × PC | 1.73 | .195 | .04 |  | O × PC | 1.65 | .206 | .03 |
|  | T × PC | 6.03 | .018 | .11 |  | T × PC | 1.81 | .185 | .04 |
|  | O × T × PC | 16.61 | < .001 | .26 |  | O × T × PC | 0.86 | .358 | .02 |
| 2 | O | 0.01 | .927 | .00 |  | O | 1.80 | .186 | .04 |
|  | T | 4.46 | .040 | .09 |  | T | 1.99 | .165 | .04 |
|  | PC | 21.93 | < .001 | .32 |  | PC | 39.82 | < .001 | .46 |
|  | O × T | 13.17 | < .001 | .22 |  | O × T | 0.55 | .460 | .01 |
|  | O × PC | 0.32 | .573 | .01 |  | O × PC | 0.24 | .629 | .01 |
|  | T × PC | 3.45 | .070 | .07 |  | T × PC | 0.10 | .760 | .00 |
|  | O × T × PC | 3.85 | .056 | .08 |  | O × T × PC | 0.25 | .617 | .01 |
| 3.1 | O | 0.17 | .681 | .00 |  | O | 0.86 | .358 | .02 |
|  | T | 1.11 | .297 | .02 |  | T | 3.21 | .080 | .06 |
|  | PC | 51.30 | < .001 | .52 |  | PC | 1.86 | .180 | .04 |
|  | O × T | 10.48 | .002 | .18 |  | O × T | 7.08 | .011 | .13 |
|  | O × PC | 0.43 | .515 | .01 |  | O × PC | 3.20 | .080 | .06 |
|  | T × PC | 3.19 | .080 | .06 |  | T × PC | 2.53 | .118 | .05 |
|  | O × T × PC | 19.61 | < .001 | .29 |  | O × T × PC | 2.04 | .160 | .04 |
| 3.2 | O | 4.06 | .050 | .08 |  | O | 0.00 | .952 | .00 |
|  | T | 1.22 | .276 | .03 |  | T | 1.66 | .204 | .03 |
|  | PC | 0.28 | .596 | .01 |  | PC | 1.26 | .267 | .03 |
|  | O × T | 7.78 | .008 | .14 |  | O × T | 2.73 | .105 | .06 |
|  | O × PC | 0.10 | .755 | .00 |  | O × PC | 0.02 | .899 | .00 |
|  | T × PC | 0.31 | .578 | .01 |  | T × PC | 0.08 | .781 | .00 |
|  | O × T × PC | 0.02 | .883 | .00 |  | O × T × PC | 0.00 | .968 | .00 |
| 4.1 | O | 0.23 | .637 | .01 |  | O | 0.27 | .607 | .01 |
|  | T | 16.82 | < .001 | .29 |  | T | 0.51 | .479 | .01 |
|  | PC | 78.79 | < .001 | .65 |  | PC | 30.21 | < .001 | .42 |
|  | O × T | 0.41 | .523 | .01 |  | O × T | 4.39 | .042 | .10 |
|  | O × PC | 6.25 | .016 | .13 |  | O × PC | 0.15 | .704 | .00 |
|  | T × PC | 13.58 | < .001 | .24 |  | T × PC | 7.49 | .009 | .15 |
|  | O × T × PC | 0.07 | .789 | .00 |  | O × T × PC | 0.95 | .335 | .02 |
| 4.2 | O | 1.69 | .200 | .04 |  | O | 0.45 | .507 | .01 |
|  | T | 1.68 | .202 | .04 |  | T | 1.31 | .259 | .03 |
|  | PC | 15.60 | < .001 | .27 |  | PC | 0.11 | .746 | .00 |
|  | O × T | 0.16 | .690 | .00 |  | O × T | 0.44 | .510 | .01 |
|  | O × PC | 10.17 | .003 | .20 |  | O × PC | 0.61 | .438 | .01 |
|  | T × PC | 6.98 | .012 | .14 |  | T × PC | 0.34 | .564 | .01 |
|  | O × T × PC | 1.14 | .293 | .03 |  | O × T × PC | 0.01 | .924 | .00 |

*Note*. The number within Exp. represents the corresponding experiment, with Experiments 3 and 4 subdivided into 3.1/4.1 for biased items and 3.2/4.2 for unbiased items; Exp. = Experiment; RT = response time; ERR = error rate; O = Order of Transition (MC-MI first, MI-MC first); T = Transition (MC-MI, MI-MC); PC = Proportion Congruency (MC, MI); I = Item Type (biased, unbiased); CE = congruency effect

# Appendix B

# Face-name combinations of Experiment 4

Fig. B1 Face-name combinations of stimulus set 1


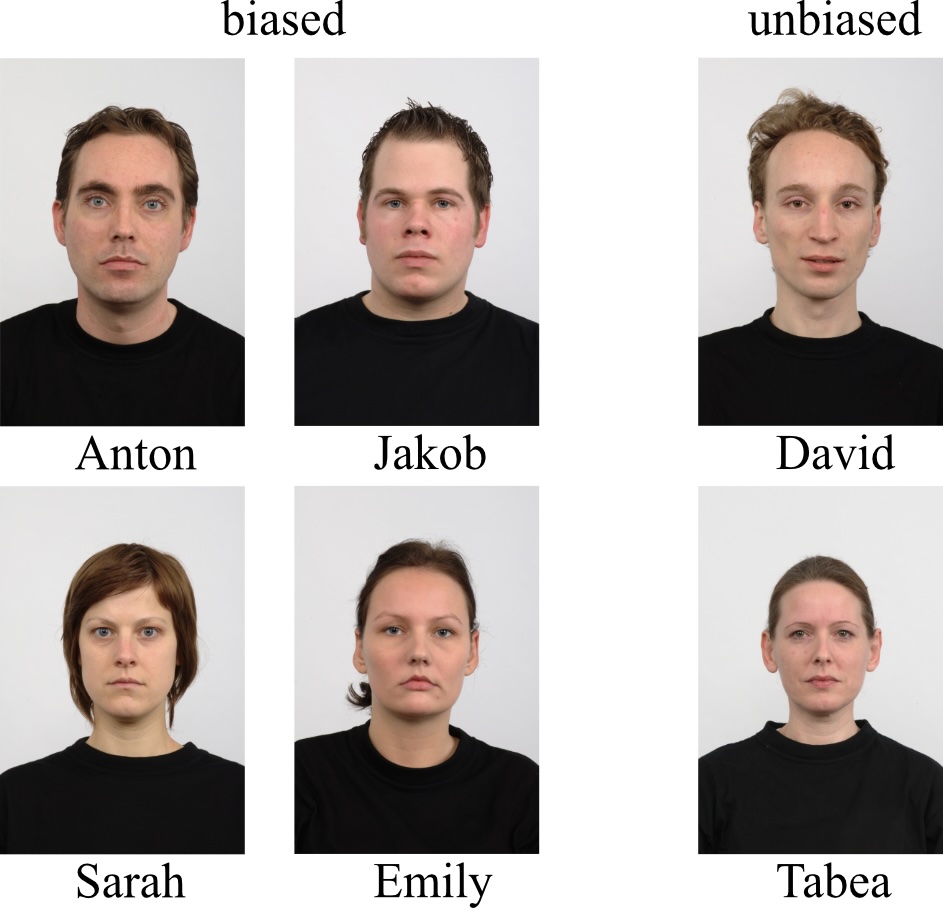


Fig. B2 Face-name combinations of stimulus set 2


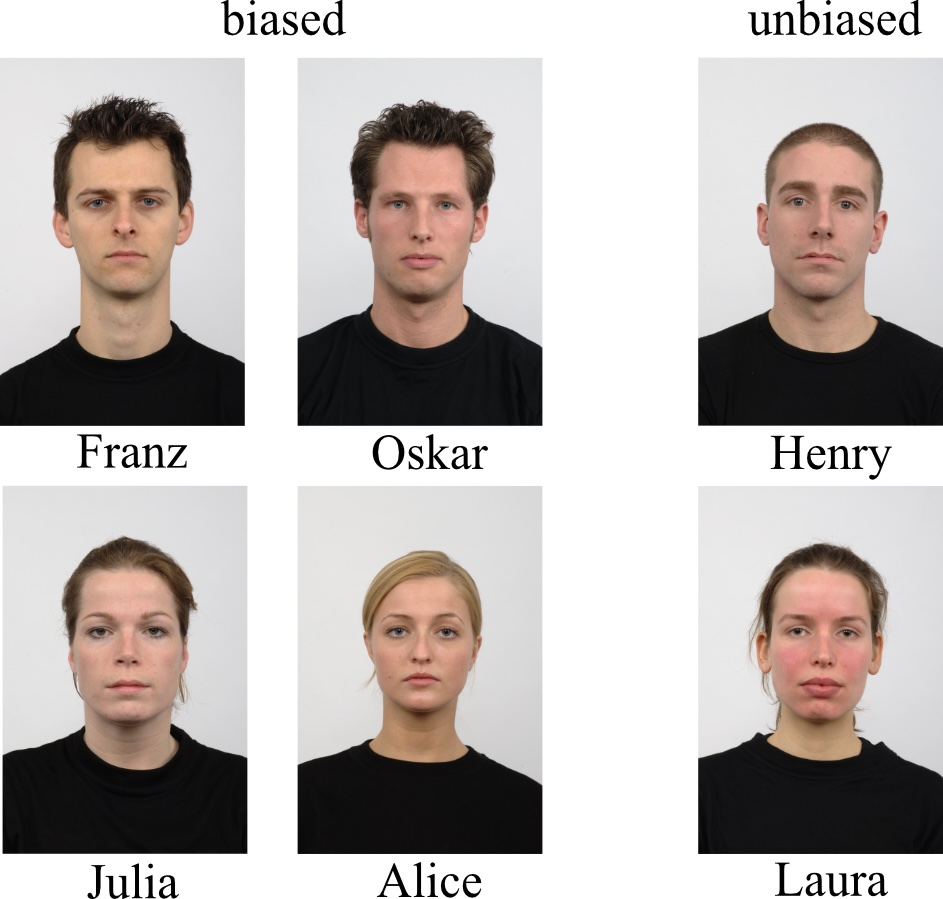

Supplement: Supplementary file 1 — Supplementary file1 (DOCX 320 KB) [file 13414_2026_3284_MOESM1_ESM.docx]
